# Quantitative Modeling Extends the Antibacterial Activity of Nitric Oxide

**DOI:** 10.3389/fphys.2020.00330

**Published:** 2020-04-17

**Authors:** Darshan M. Sivaloganathan, Mark P. Brynildsen

**Affiliations:** ^1^Program in Quantitative and Computational Biology, Princeton University, Princeton, NJ, United States; ^2^Department of Chemical and Biological Engineering, Princeton University, Princeton, NJ, United States

**Keywords:** *Escherichia coli*, Hmp, flavohemoglobin, NO, respiration, bacteriostatic

## Abstract

Numerous materials have been developed to try and harness the antimicrobial properties of nitric oxide (NO). However, the short half-life and reactivity of NO have made precise, tunable delivery difficult. As such, conventional methodologies have generally relied on donors that spontaneously release NO at different rates, and delivery profiles have largely been constrained to decaying dynamics. In recent years, the possibility of finely controlling NO release, for instance with light, has become achievable and this raises the question of how delivery dynamics influence therapeutic potential. Here we investigated this relationship using *Escherichia coli* as a model organism and an approach that incorporated both experimentation and mathematical modeling. We found that the best performing delivery mode was dependent on the NO payload, and developed a mathematical model to quantitatively dissect those observations. Those analyses suggested that the duration of respiratory inhibition was a major determinant of NO-induced growth inhibition. Inspired by this, we constructed a delivery schedule that leveraged that insight to extend the antimicrobial activity of NO far beyond what was achievable by traditional delivery dynamics. Collectively, these data and analyses suggest that the delivery dynamics of NO have a considerable impact on its ability to achieve and maintain bacteriostasis.

## Introduction

Nitric oxide (NO) is a diatomic, hydrophobic, free radical gas with a wide array of antimicrobial properties (Fang, 2004; Thomas et al., 2008). When present at concentrations in the micromolar (μM) range and above, NO can directly impair enzyme activity by irreversibly damaging iron-sulfur cluster residues and inhibit cellular respiration by binding heme groups within cytochrome oxidases (Wink and Mitchell, 1998; Thomas et al., 2008; Radi, 2018). Additionally, it is capable of reacting with oxygen and superoxide spontaneously to generate even more deleterious species that can cause protein damage, through thiol and tyrosine nitrosylation, DNA damage through base deamination, and damage to membranes and lipid structures through lipid peroxidation (Hogg and Kalyanaraman, 1999; O’Donnell and Freeman, 2001; Vázquez-Torres and Fang, 2005; Toledo and Augusto, 2012). These diverse cytotoxic effects can ultimately impair bacterial metabolism, inhibit growth, and cause cell death.

Within the context of innate immunity, phagocytic cells harness NO to combat and eliminate invading pathogens (Fang, 2004; Flannagan et al., 2009; Henard and Vázquez-Torres, 2011). The importance of NO to pathogen virulence has been demonstrated by the large number of bacteria that require NO detoxification systems for survival (Poole and Hughes, 2000; Poole, 2005). For example, *Salmonella enterica* lacking the flavohemoglobin Hmp were found to be more susceptible to killing by macrophages (Stevanin et al., 2002; Bang et al., 2006). Similarly, deletion of Hmp in uropathogenic *E. coli* was found to significantly impair its ability to colonize the urinary tract (Svensson et al., 2010). In addition, the inability to produce NO in a murine model, through deletion of inducible nitric oxide synthase (iNOS), has been linked to increased likelihood of infection by *Mycobacterium tuberculosis*, *Listeria monocytogenes*, and *Leishmania* spp. (MacMicking et al., 1995). Conversely, increased iNOS expression has been associated with reduced malaria symptoms, as well as the decreased possibility of relapse (Kun et al., 2001; Hobbs et al., 2002).

The potent and broad-spectrum antimicrobial properties of NO have led to the development of numerous NO therapeutics (Kim et al., 2014; Yang et al., 2015). Many small chemical compounds and functional moieties have been developed to exogenously produce NO in response to heat, pH, and enzymatic catalysis. Some of the most widely used and studied NO-releasing moieties include diazeniumdiolates (NONOates) and S-nitrosothiols (Riccio and Schoenfisch, 2012; Sadrearhami et al., 2018). In recent years, different materials and delivery vehicles have been designed to take advantage of the release properties of these chemistries. Polymer scaffolds, gels, and coatings represent one large class of such materials (Kim et al., 2014; Liang et al., 2015). Examples include NO-releasing polymer coatings (Ho et al., 2017), NO-releasing sol-gels (Nablo et al., 2005), and NO-releasing chitosan oligosaccharides (Lu et al., 2014). Ho and colleagues demonstrated that exposure of *P. aeruginosa* or *S. aureus* to NONOate coatings significantly reduce bacterial adhesion and biofilm formation (Ho et al., 2017). Moreover, NONOate based sol-gels have been evaluated as potential coatings for orthopedic devices, where coated medical grade steel was effective at inhibiting *P. aeruginosa*, *S. aureus*, and *S. epidermidis* adhesion (Nablo et al., 2005). Lu and colleagues designed NONOate-based chitosan oligosaccharides that were extremely effective at penetrating biofilms and killing *P. aeruginosa*, while providing essentially no toxicity to mouse fibroblast cells (Lu et al., 2014). Another significant class of NO delivery vehicles are nanoparticles (Quinn et al., 2015). Kafshgari and coworkers devised porous silica-based nanoparticles conjugated to S-nitrosothiols and S-nitrosogluthatione and showed that they have significant antimicrobial activity against *E. coli* and *S. aureus* (Hasanzadeh Kafshgari et al., 2016). Overall, there has been sustained, growing interest in developing NO materials and delivery vehicles capable of harnessing the antimicrobial properties of NO. The examples mentioned above represent only a fraction of such compounds.

Despite the development of numerous NO materials, few have been evaluated for therapeutic purposes or have translated to clinical settings (Liang et al., 2015; Yang et al., 2015). One of the issues is associated with poor control of NO release. Low stability and rapid release of NO make it difficult to deliver NO for extended periods of time, maintain concentrations within desirable ranges, and provide tissue-specific activity. Traditional materials are loaded with a payload of NO donor that spontaneously dissociates when exposed to water or other conditions. As such, NO dynamics have largely been constrained to rapid accumulation of NO at the onset of delivery followed by progressive decay. Not only are these dynamics restricted, but they are in stark contrast to the way NO is delivered naturally within phagosomes. During an immune response, NO is delivered for extended periods of time, in which the rates of NO delivery have been suggested to peak hours after phagocytosis (Reichner et al., 1999; Vazquez-Torres et al., 2000; Pfeiffer et al., 2001). Recently, our group established a relationship between bolus payload and release kinetics, where at lower payloads faster dissociation rates led to greater antimicrobial activity, while at higher payloads slower dissociation rates were favored (Robinson et al., 2014b). However, the restricted set of delivery dynamics evaluated and their discordance with the way NO is delivered in physiological environments, raises the question of how this design criterion may impact the development of future NO-based therapeutics.

In recent years, the possibility of finely controlling delivery has become achievable with the development of light controlled, photoactivated compounds (Sortino, 2010; Choi et al., 2016; Sadrearhami et al., 2018). In particular, metal-nitrosyl complexes have gained significant attention, as alternative NO releasing moieties, because of their ability to induce NO release upon exposure to specific wavelengths of light (Tfouni et al., 2012; Xiang et al., 2017). The Mascharak group developed manganese-nitrosyl sol-gel coatings that released NO upon exposure to near infrared light (NIR) and led to significant reduction of *S. aureus*, *E. coli* and *A. baumannii* bacterial loads (Heilman and Mascharak, 2013). Similarly, Evans and colleagues developed manganese-nitrosyl based polymer microparticles that release NO upon exposure to NIR (Evans et al., 2018). Roveda and coworkers designed polyamidoamine dendrimers modified with ruthenium nitrosyl moieties, which could be activated upon UV irradiation (Roveda et al., 2014). In addition to light-activated compounds, enzymatic pro-drug systems represent another methodology to finely tune delivery rates through the control of enzymes or substrates. Jones and colleagues developed a NO probiotic patch in which *Lactobacilli* fermentation of glucose lead to NO production from nitrite (Jones et al., 2010). The Zhao group generated a unique methylated galactose NONOate conjugate that was only recognizable by a mutant beta galactosidase enzyme from *Thermus thermophilus* (Hou et al., 2019). NONOate release was restricted to environments containing the selective beta galactosidase and by co-delivering the enzyme and pro-drug, which allowed localization of NO release to specific tissues and reduced systemic toxicity.

The capability of precisely controlling NO delivery raises several interesting questions, such as, how delivery dynamics influence the antimicrobial potency of NO; and what is the best way to deliver a given payload of NO? To begin to address these questions, we used an approach that integrated experiments and computational modeling to assess, analyze, and predict how NO delivery dynamics influence the duration of nitrosative stress in *E. coli* cultures. Using fed-batch bioreactors, we evaluated four basic modes of delivery, one of which was a traditional bolus delivery, and observed that dosing outcome differed drastically depending on the payload administered. That data was used to train a computational model of the *E. coli* NO stress network, which was able to accurately predict the NO concentration profiles and clearance times when larger payloads were administered. Quantitative analysis of those results suggested that maintaining respiratory inhibition was a major driver of delivery outcome, which was a prediction confirmed by further experimentation. Finally, with the model as a guide, we constructed delivery regimes capable of maintaining steady state NO concentrations at levels sufficient to inhibit cellular respiration, and this led to dosing schedules that were far more effective than any other tested delivery schemes. Collectively, the data and analyses presented here demonstrate the importance of dosing dynamics when designing NO-based treatments.

## Results

### Bioreactor Configuration to Modulate NONOate Delivery

**FIGURE 1:**
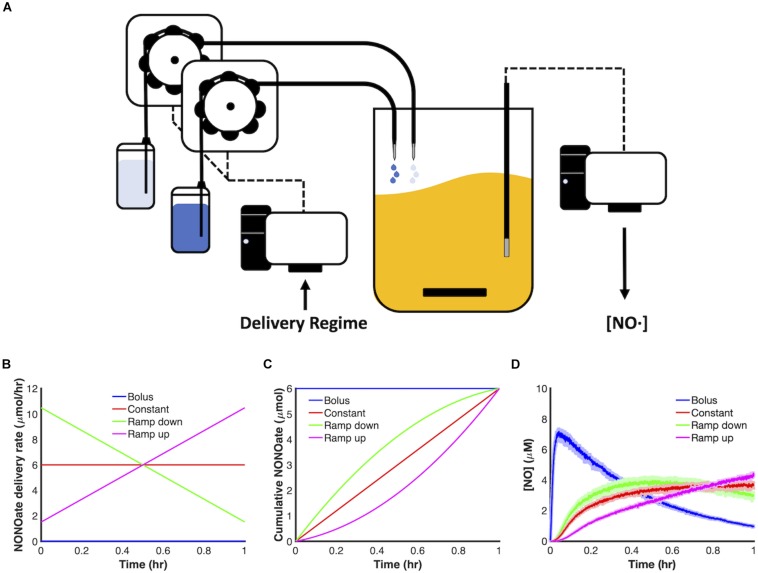
Control of NONOate delivery dynamics. **(A)** Schematic of a computer-controlled, fed-batch bioreactor used to deliver NONOate. Delivery schedules were programmed using a computer-controlled low flow drip system. Once initiated, individual peristaltic pumps drew solutions from two reservoirs: PAPA NONOate solution (dark blue) and 10 mM NaOH (light blue), which then dripped into a bioreactor containing 50 mL MOPS minimal media. [NO] was continuously measured in the bioreactor using an electrochemical probe. **(B,C)** 6 μmol PAPA NONOate was delivered over an hour in four modes: bolus (blue), constant (red), ramp down (green), ramp up (pink). **(D)** Measured [NO] dynamics, for each mode, during delivery of 6 μmol PAPA NONOate over an hour in the absence of cells. Solid lines represent the mean of three replicates, whereas the lightly shaded areas represent the standard error of the mean.

In this study, we sought to investigate the impact of delivery dynamics on the antimicrobial potency of NO. To do this, we constructed a system capable of finely tuning delivery of NO releasing compounds (NONOates) (Figure 1A). Specifically, our system is composed of a fed-batch bioreactor, in which the input flowrates of NONOate and its balance stream (NONOate solvent) can be programmed and automated using a low flow control system. We elected a drip system to eliminate the possibility of back flow, which was a concern due to the low flowrates we planned to use (as low as 10 μL/min). We are able to measure and monitor several outputs, such as the concentration of NO and O_2_ present in the bioreactor, as well as temperature and culture turbidity.

We chose to begin our investigation by evaluating four principle modes of delivery. In particular, we examined the dynamics of linearly increasing (ramp up), linearly decreasing (ramp down) and constant delivery regimes and compared them to the traditional delivery method, which is a bolus (Figure 1B). Delivery schemes were implemented over 1 h with a total payload of 6 μmol PAPA NONOate delivered (Figure 1C). To maintain identical volumes with the different schema as a function of time, a secondary drip system delivered a balance stream, which was 10 mM NaOH (solvent for PAPA NONOate). In the control case of bolus delivery, both reservoirs were programmed to deliver 10 mM NaOH over 1 h. Figure 1D depicts the differing NO dynamics in cell-free systems for these four modes of delivery.

### Type of Delivery Mode Influences the Duration of NO Stress in a Payload-Dependent Manner

To begin exploring NO detoxification under different delivery schema, aerobic cultures of *E. coli* were grown to mid-exponential phase and inoculated into a bioreactor at an optical density at 600 nm (OD_600_) of 0.05 before being treated with 6 μmol of PAPA NONOate, delivered using each of the four modes, over 1 h, with the exception of bolus which was introduced at the onset. Our metric of interest to evaluate different delivery modes is NO clearance time (t_clear_), which is the time during which the concentration of NO ([NO]) is greater than or equal to 0.5 μM. This concentration was chosen because NO at μM concentrations or above exerts nitrosative stress (Thomas et al., 2008).

**FIGURE 2:**
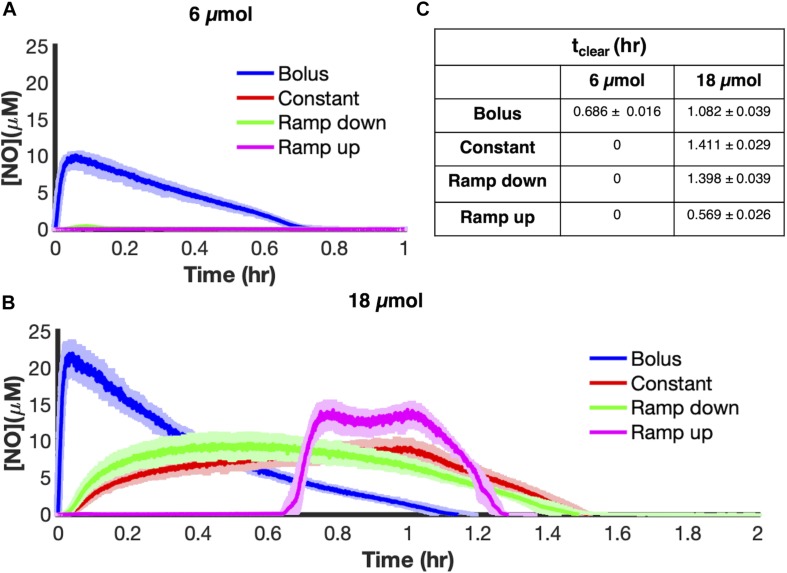
Delivery outcome is payload dependent. *E. coli* cultures were grown to exponential phase and inoculated in a bioreactor at an OD_600_ of 0.05. Five minutes after inoculation, PAPA NONOate was delivered over an hour in one of four ways (bolus-blue; constant-red; ramp down-green; ramp up-pink) at payloads of **(A)** 6 μmol or **(B)** 18 μmol. [NO] was measured continuously using an ISO-NOP probe. Solid lines represent the mean of three independent experiments, whereas the lightly shaded areas represent the standard error of the mean. **(C)** The duration of nitrosative stress (t_clear_) was measured at 6 μmol and 18 μmol for each delivery scheme (calculated as the time for which [NO] ≥ 0.5 μM). Values represent the average value ± the standard error of the mean.

At a payload of 6 μmol PAPA NONOate, bolus delivery led to an [NO] peak of 10.32 ± 0.37 μM (Figure 2A) and NO was cleared from the culture by 0.686 ± 0.016 h. In contrast, the other delivery schemes failed to reach 0.5 μM, and thus did not result in nitrosative stress. Interestingly, dosing higher payloads (18 μmol), led to strikingly different dynamics (Figure 2B). All four delivery schema produced nitrosative stress, with constant delivery being the most effective dosing scheme with an NO clearance time of 1.411 ± 0.029 h, which was a thirty percent increase in t_clear_ compared to bolus delivery of the same payload. This result suggested that the ability of NO to cause nitrosative stress depends both on the payload and the dynamics of how it is delivered.

### Computational Modeling of NO Stress

To quantitatively explore the relationship between delivery dynamics and antimicrobial efficacy, we trained a kinetic model of NO stress in *E. coli* using the data obtained at 6 and 18 μmol. The model was developed in previous studies (Robinson and Brynildsen, 2013, 2015, 2016a,b; Robinson et al., 2014a, b; Sacco et al., 2017) and expanded upon here. Specifically, the model was adjusted to comply with fed-batch systems and cellular growth was incorporated and assumed to depend on the availability of aerobic cytochrome oxidases for respiration. Uncertain parameters were trained using a non-linear least squared optimization algorithm, followed by a Markov Chain Monte Carlo (MCMC) procedure. Parameter sets were accepted based on Evidence Ratios (ER) and ensembles of models were generated (section Materials and Methods). A complete list of species, reactions, and kinetic parameters can be found in Supplementary Tables S1–S3.

#### Model Adjustments for Fed-Batch Operation

To simulate our microfluidic drip system, continuous NONOate delivery and extracellular species dilution were incorporated into an existing kinetic model of NO metabolism (Robinson and Brynildsen, 2016b). Specifically, an input term was added to the rate equation for the NONOate species balance to capture influx of NONOate. The input term had four functional forms, depending on the delivery mode implemented (section Materials and Methods and Supplementary Methods). A volume dependent dilution term was also included to capture dilution of extracellular species, as a result of volume expansion within the bioreactor during operation (section Materials and Methods and Supplementary Methods).

#### Incorporation of Cellular Growth

Previous iterations of the model used in this study did not account for cellular growth but rather focused on the period of NO stress. This was done because NO is bacteriostatic, and thus under NO stress cells are non-growing. However, as depicted for three of the 6 μmol delivery modes (constant, ramp up, ramp down) and one of the 18 μmol schemes (ramp up), long periods of time without NO stress were present, and OD_600_ measurements revealed that cells were growing during those periods (Supplementary Figures S1B,D). Growth rate was modeled as a 1st order Hill-type function.

(1) $$\mu =\mu_{m\,a\,x}\cdot \frac{\left[C\,y\,t\,o\,c\,h\,r\,o\,m\,e_{b\,o}\right]+\left[C\,y\,t\,o\,c\,h\,r\,o\,m\,e_{b\,d}\right]}{K_{\mu }+\left[C\,y\,t\,o\,c\,h\,r\,o\,m\,e_{b\,o}\right]+\left[C\,y\,t\,o\,c\,h\,r\,o\,m\,e_{b\,d}\right]}$$

Where μ_*max*_ is the maximum specific growth rate and *K*_μ_ represents the concentration of cytochromes required to reach half the maximum growth specific rate. Under aerobic conditions, the majority of ATP production in *E. coli* is accounted for by cellular respiration (Baron, 1996; Trotter et al., 2011; Soria et al., 2013) and therefore we chose to define the specific growth rate equation as a function of freely available terminal cytochrome *bo* and *bd*-I oxidases. A set of 16 uncertain respiratory parameters (section Materials and Methods and Supplementary Table S5), were trained on [O_2_] and OD_600_ data obtained from aerobic, mid-exponential phase *E. coli* treated with three concentrations of KCN (0, 50, and 1000 μM) (Supplementary Figure S2). The ensemble of models could accurately capture O_2_ consumption and cell density at all three concentrations of KCN. Additionally, growth-dependent dilution terms were incorporated into rate equations for cellular species to capture the expansion of intracellular volume that occurs with growth (section Materials and Methods). Further, in previous iterations (Robinson and Brynildsen, 2016b) the protein translation rate was modeled as a function of [O_2_], a reflection of energy production through O_2_ consumption by terminal cytochromes; however, here we adjusted that rate expression so that translation was directly related to growth (Materials & Methods).

#### Model Training and Experimental Validation

**FIGURE 3:**
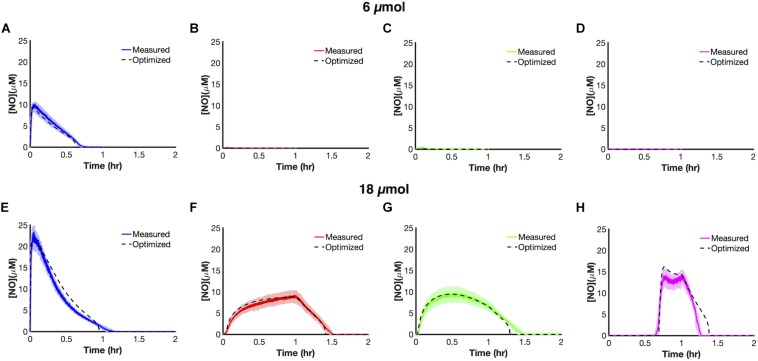
Model training and optimization on NO dynamics observed at 6 μmol **(A–D)** and 18 μmol **(E–H)** for the four principle dosing modes (refer to Supplementary Table S6 for a list of optimized parameters). Cultures of *E. coli* were grown to exponential phase and inoculated in a bioreactor at an OD_600_ of 0.05. Five minutes after inoculation, PAPA NONOate was delivered over an hour in one of four ways (bolus- blue; constant-red; ramp down-green; ramp up-pink). Solid lines represent the mean of three independent experiments, whereas the lightly shaded areas represent the standard error of the mean. Dashed black lines represent simulation results using the ensemble of parameter sets (ER < 10, 28 sets in total) trained on the data presented in this figure. Simulations from the ensemble members greatly overlapped, thus resembling a single line.

**FIGURE 4:**
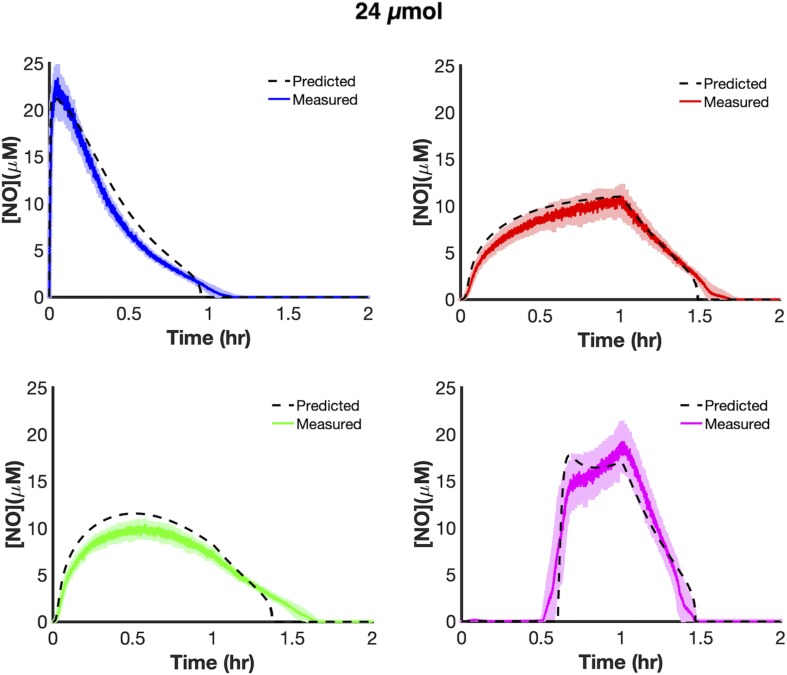
Model extrapolation and predictions at 24 μmol payloads. Dashed black lines represent predicted [NO] dynamics using the ensemble of parameter sets (ER < 10, 28 sets in total). Simulations from the ensemble members greatly overlapped, thus resembling a single line. Colored lines represent measured [NO] dynamics (bolus- blue; constant-red; ramp down-green; ramp up-pink). The solid lines represent the mean of three independent experiments, whereas the lightly shaded areas represent the standard error of the mean.

We trained uncertain parameters related to cellular NO consumption on all [NO] and [O_2_] data measured at 6 and 18 μmol (21 parameters in total, Supplementary Table S6). Simulations for the ensembles of models did a good job of capturing data at both 6 and 18 μmol for the different delivery modes (Figure 3). To assess the utility of the model, we tested its predictive power by simulating each delivery mode at 24 μmol. The model predicted that bolus delivery should lead to a t_clear_ of 0.957 h and that it would be outcompeted by ramp down and constant modes, with t_clear_ of 1.359 and 1.462 h, respectively, whereas it should still be more effective than ramp up with a t_clear_ of 0.859 h. Experimental measurements agreed well with those forward predictions from the model (Figure 4). This confirmed that the model could accurately extrapolate to conditions outside its training data, which gave confidence that it could be used to quantitatively analyze NO stress in *E. coli* cultures.

### Evaluating NO Clearance by Varying the Delivery Time

**FIGURE 5:**
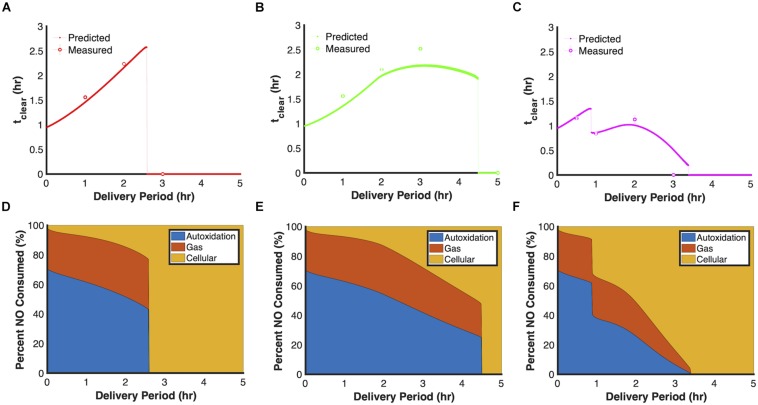
Relationship between t_clear_ and delivery period. Model simulations using the ensemble of parameter sets (ER < 10, 28 sets in total) were performed by delivering 24 μmol PAPA NONOate and varying the delivery period between 0 and 5 h and calculating t_clear_ for each simulation. **(A)** Constant, **(B)** ramp down, **(C)** ramp up. Solid lines represent predicted relationship between t_clear_ and delivery period, while dashed lines represent discontinuities in the curves. Circles represent mean t_clear_ values from at least three experiments and error bars represent the standard error of the mean. Predicted NO cumulative distribution profiles using the optimal parameter set (ER = 1, minimum SSR, 1 set) up to the end of the delivery period or when [NO] dropped below 0.5 μM, whichever was greater for constant **(D)**, ramp-down **(E),** and ramp-up **(F)** delivery schedules. The three major NO consumption pathways are autoxidation (blue), transport to gas phase (red), and cellular consumption (yellow).

We sought to evaluate the dynamics of three of the principle dosing modes by varying an additional parameter, duration of delivery. The analysis focused on 24 μmol payloads and the total time to achieve that dosage. As depicted in Figures 5A–C, extending the delivery period lengthened t_clear_ for constant (red trend line) and ramp-down (green trend line) delivery modes to such an extent that their t_clear_ exceeded that of bolus delivery (t_clear_ = 0.957 h) by more than twofold, whereas the most effective delivery periods for ramp up (purple trend line) were less than an hour. In addition, simulations revealed that each delivery mode displayed distinct discontinuities when plotting t_clear_ against delivery period. Evaluation of the cumulative NO consumption flux profiles (Figures 5D–F), suggested that the discontinuities were associated with failures to inhibit cellular respiration, which led to higher translation rates and ultimately higher concentrations of Hmp (Supplementary Figures S3–S5), which is the main NO detoxification enzyme under aerobic conditions (Gardner and Gardner, 2002; Corker and Poole, 2003; Robinson and Brynildsen, 2013, 2016b). Noticeably, the ramp-up delivery mode contains two discontinuities, where the first was due to an initial failure to inhibit cellular respiration which allowed increased translation and Hmp protein expression. This led to cellular NO consumption rates that balanced NO delivery rates. However, near the end of the delivery period, the increasing delivery rates began to exceed cellular consumption, which led to a sudden rise in [NO]. While the second discontinuity, was similarly due to a failure to inhibit cellular respiration, and cellular consumption invariably balanced NO influx throughout delivery. Experiments were performed to assess the accuracy of these predictions, and as depicted by the colored dots in Figures 5A–C, data agreed well with model predictions, including the approximate delivery times that corresponded to the discontinuities.

**FIGURE 6:**
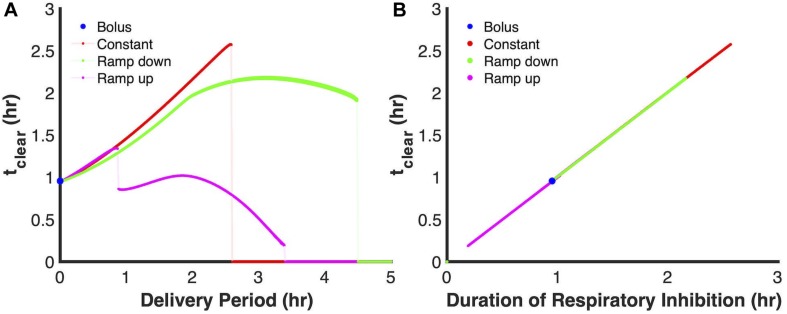
Duration of respiratory inhibition is a strong predictor of t_clear_. **(A)** Model predictions using the ensemble of parameter sets (ER < 10, 28 sets in total) for t_clear_ as a function of delivery period when a 24 μmol PAPA NONOate payload is delivered. Solid lines represent the predicted relationship between t_clear_ and delivery period (bolus- blue; constant-red; ramp down-green; ramp up-pink), while the lightly shaded lines represent discontinuities in the curves. **(B)** Plot of duration for respiratory inhibition vs. t_clear_. Duration of respiratory inhibition was defined as the length of time for which the percentage of NO bound cytochromes ≥ 99%.

Given the central role of respiratory inhibition in defining the delivery periods at which the principle modes become ineffective (or less effective for the first discontinuity of the ramp up mode), we plotted t_clear_ as a function of the duration during which respiration is inhibited. We considered respiratory inhibition, as the time for which 99% or more of terminal cytochrome oxidase were NO bound. As depicted in Figure 6, all of the simulations, regardless of delivery mode, fall onto a single line. This suggested that the duration of NO stress is strongly associated with the ability to achieve and maintain respiratory inhibition.

### Maintaining Respiratory Inhibition to Maximize the Duration of NO Stress

**FIGURE 7:**
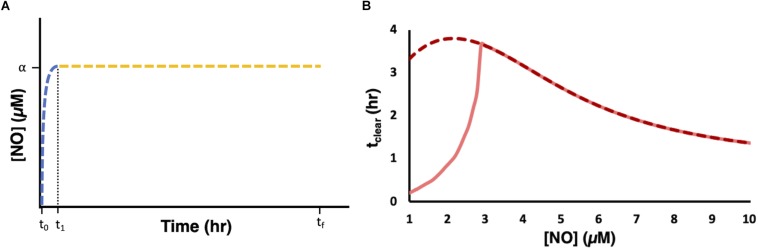
Designing and implementing delivery schemes to maintain steady state [NO]. **(A)** For a desired NO concentration α and payload ω, a bolus delivery was introduced at t_0_ such that the [NO] profile (blue dashed line) peaked at [NO] = α at t_1_. At t_1_ a dosing regime was implemented to maintain *d*[*NO*]/*dt* = 0 and [NO] = α up until t_*f*_ (yellow dashed line), at which point the payload ω was exhausted. **(B)** Steady state dosing regimes were simulated at [NO] between 1 and 10 μM for a payload of 24 μmol and t_clear_ was measured (dark red dashed line). The light shaded solid red line represent the relationship between t_clear_ and [NO] when implementing a step function delivery approximation. All simulations were performed using the optimal parameter set (ER = 1, minimum SSR, 1 set).

**FIGURE 8:**
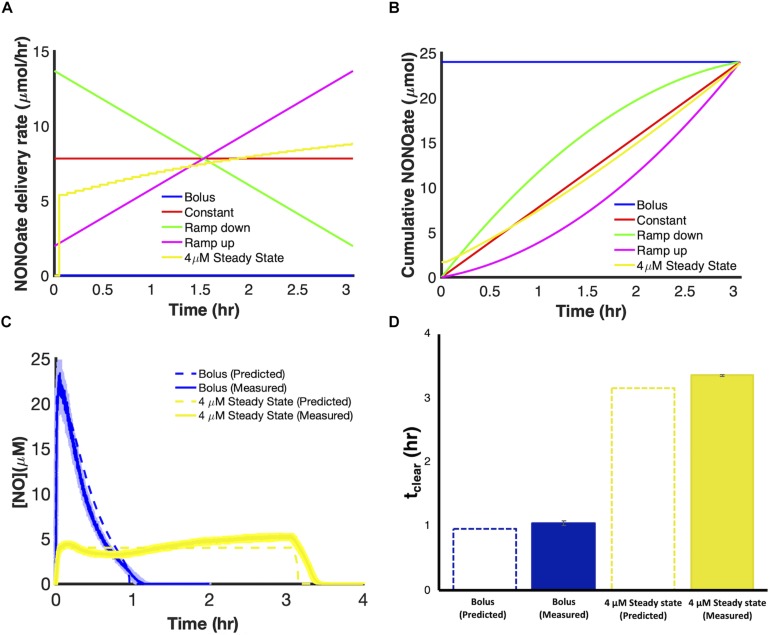
Implementation of a delivery schedule to maintain [NO] at 4 μM steady state with a payload of 24 μmol. Comparison of delivery rate **(A)** and cumulative NONOate **(B)** profiles for a 4 μM steady state dosing schedule and the principle delivery modes over identical delivery periods. Comparison of the predicted and measured [NO] profiles **(C)** and t_clear_ values **(D)** for a 4 μM steady state dosing scheme compared to a bolus delivery. Dashed colored lines represent predicted [NO] dynamics using the optimal parameter set (ER = 1, minimum SSR, 1 set). Solid colored lines represent the mean of three independent experiments, while lightly shaded areas represent the standard error of the mean.

We used the model to evaluate the relationship between [NO] and respiratory inhibition and found that NO concentrations slightly above 1 μM (∼1.2 μM) corresponded to 99% NO bound cytochrome (Supplementary Figure S6). We hypothesized that, for a given payload, a dosing regimen that could raise and maintain NO at concentrations of 1.2 μM or greater, would extend t_clear_ beyond that which could be achieved with bolus administration or any of the principal modes. Using the model, we designed delivery schema capable of maintaining steady state concentrations of NO. Specifically, this was accomplished by constructing composite delivery schemes (Figure 7A). First, a bolus was introduced to raise NO to the desired steady state concentration. Then when [NO] had reached its peak value a dosing scheme was solved for, using the remainder of the payload, to deliver NO at a rate that balanced NO consumption, as predicted by the model, and maintain d[NO]/dt equal to zero. Composite delivery schemes were designed in this manner for various concentrations of NO greater than or equal to 1 μM. The model predicted that the optimal composite dosing regime was achieved by maintaining NO at approximately 2.2 μM. Model simulations suggested that a bolus payload of 0.8 μmol would lead to an NO profile that peaked at 2.2 μM and that implementing a dosing schedule to maintain NO at 2.2 μM, with the remaining 23.2 μmol, could extend t_clear_ to over 3.8 h. Experimental application of the composite dosing regimen failed to recapitulate the predicted NO dynamic (Supplementary Figure S7A), and severely underperformed (t_clear_ = 0.2 h) compared to the predicted t_clear_. A deeper analysis revealed that this inaccurate prediction was due to physical limitations of our experimental system. Specifically, the pumping system required us to approximate dosing schedules with piecewise step functions (Supplementary Figure S7B). Taking into account this source of error, the model predicted that t_clear_ was not robust to these variations until the steady NO concentration exceeded approximately 3 μM (Figure 7B). Therefore, we chose to implement a delivery regime to maintain [NO] at 4 μM, which is well within the regime where simulations with the piece-wise step function agree well with the continuous delivery function. Simulations revealed that a 4 μM [NO] peak was achieved by a bolus payload of 1.7 μmol. The remaining 22.3 μmol were delivered to maintain [NO] at 4 μM (Figures 8A,B). Simulations suggested that it was possible to extend t_clear_ to over 3 h by delivering in this manner, which would be over threefold higher than bolus administration of 24 μmol. The delivery scheme was implemented experimentally and the measured [NO] profile agreed well with simulations (Figure 8C). This dosing schedule proved to be far more effective than any of the principle delivery modes, and more specifically, it led to a threefold increase in t_clear_ when compared to a bolus delivery of the same payload (Figure 8D).

## Discussion

Nitric oxide is a potent antibacterial harnessed by macrophages of the innate immune response (Radtke and O’Riordan, 2006; Haas, 2007; Bowman et al., 2011). The potential of NO as an antimicrobial treatment has led to the development of numerous materials capable of directly delivering NO to infection sites (Seabra and Durán, 2010; Schairer et al., 2012a). For example, Martinez and coworkers demonstrated that delivery of silica-based nanoparticle into skin lesions of MRSA-infected mice, led to significant reductions of bacterial burden when compared to untreated infections (Martinez et al., 2009). Nablo and colleagues developed silicone elastomer implants coated with NONOate sol-gels that led to an 82% reduction in the number of *S. aureus* infected implants when compared to uncoated implants in a rat model (Nablo et al., 2005). Notably, these animal studies were conducted with materials that would spontaneously release NO with a decaying rate. The limited control over NO release has led to restricted NO dynamics, in which NO profiles exhibit high initial levels that decline as a function of time. Given the advent of materials with increasingly tunable NO delivery, such as photo controllable and enzyme pro-drug systems, the question of whether other modalities of NO release could influence therapeutic outcomes arises.

We began by constructing a system capable of tuning NO delivery and measured how cultures of *E. coli* responded to treatment. In particular, we explored three primary modes of delivery (linearly increasing, decreasing, and constant modes) and compared them to bolus. At lower payloads, we observed that bolus delivery was the only effective method. While at higher payloads delivery outcome was quite different with all four delivery schedules providing periods of nitrosative stress (t_clear_) and two of the four outcompeting bolus. With the observation that the efficacies of delivery regimens were a function of payload, we sought to develop a computational model that could predict NO dynamics under different delivery scenarios. Using the data obtained at both payloads (6 and 18 μmol), we trained a model of the NO biochemical network and showed that the model was effective at extrapolating to higher payloads (24 μmol) and predicting the outcome.

We continued our analysis by exploring how delivery period, as a variable, influenced antimicrobial activity. The model predicted that by extending the delivery period it was possible to extend t_clear_ to be greater than twofold of a bolus of the same payload. Moreover, the model predicted sudden changes in t_clear_, for each principle mode, as the delivery period was extended beyond specific thresholds. The model predicted that these sudden changes were due to delivery rates that failed to inhibit cellular respiration and thereby led to increased Hmp protein expression in growing cells. Experiments confirmed the trends predicted by the model, which led us to define a metric representative of the length of time under respiratory inhibition. A model-facilitated analysis revealed that the longer cells were unable to respire, the longer it took cells to detoxify NO, and when t_clear_ was plotted against the duration of respiratory inhibition, all of the delivery modalities collapsed onto a single line. This led us to hypothesize that dosing regimens that maintained respiratory inhibiting concentrations of NO, for as long as possible, would extend dose efficacy beyond what we observed with the principal modes. To test this hypothesis, we used the model to design dosing schedules capable of maintaining NO concentrations at and above the threshold to inhibit cellular respiration. When we tested predictions that maintained NO at 4 μM, we were able to extend dose efficacy to over threefold what it would have been with a bolus administration of the same payload. Further, that dosing schedule also outperformed all of the other principal modes at that payload. Looking forward, it is worth noting that future work to extend bacterial NO stress could benefit from formulating the task as an optimization problem. As a first pass at this, we considered delivery schedules that conformed to third order polynomials and used an optimization algorithm to identify coefficients that maximized the amount of time cultures were exposed to NO concentrations that were inhibitory to respiration (Materials and Methods). Although that attempt at optimization did not yield solutions better than the steady-state approach we presented, there are many different ways to formulate an optimization problem and numerous algorithms to identify best solutions. We believe that future work on optimization frameworks with this application could reveal novel strategies that outperform the methodologies used in the present study.

The data presented in this study suggest that the dosing method of NO can have a significant impact on its antibacterial capabilities. Further, this work suggests that maintaining NO concentrations at levels that inhibit cellular respiration is a critical parameter for inhibiting the propagation of *E. coli* under aerobic conditions, such as those found in the urinary tract (Svensson et al., 2010; Spiro et al., 2015). Many microbes contain similar detoxification networks, generate protein homologs similar to Hmp, and thrive under oxygenated conditions (Gardner, 2005, 2012; Stern and Zhu, 2014). Therefore, inhibiting cytochrome oxidase activity may be an important variable to maximize dose efficacy of NO-releasing materials across a wide range of bacteria. We envision that such knowledge could be employed with feed-back control devices that maintain local NO levels at infection sites, such as dermal wounds, at concentrations that yield respiratory inhibition. Such delivery platforms could be important for the eventual application of these materials since NO is also deleterious to mammalian cells and there is a restricted concentration window where it is antibacterial and non-toxic to our cells, which argues against the use of bolus delivery schemes (Hurford, 2005; Friedman et al., 2011; Schairer et al., 2012a; Sun et al., 2012). However, it should be noted that one limitation of this study is associated with its time scales, which are on the order of several hours due to constraints associated with our experimental system (Materials and Methods). With an eye toward clinical applications, time scales of 24 h or longer need to be tested in order to assess whether what was found to be important at a few hours is also important over a few days (Martinez et al., 2009; Jones et al., 2010; Schairer et al., 2012b).

## Materials and Methods

### Bacterial Strains

All experiments performed in this study were conducted with *E. coli* K-12 MG1655 (Brynildsen et al., 2013).

### Chemicals and Growth Media

Growth media used in all experiments was MOPS minimal media with 10 mM glucose as the sole carbon source. The NO donor used, (Z)-1-[N-(3-aminopropyl)-N-(n-propyl)amino]diazen-1-ium-1,2-diolate (PAPA NONOate), was dissolved in 10 mM NaOH and stored on ice during delivery. Potassium cyanide (KCN) was dissolved in autoclaved Milli-Q water at a concentration of 1 M. Luria-Bertani (LB) broth was made from dissolving LB powder (40% Tryptone, 20% Yeast extract, 40% Sodium Chloride per gram of solid) in Milli-Q water and autoclaving the solution.

### Fed-Batch Bioreactor

Sterile 250 mL conical tubes (Nunc) were used as batch bioreactors for experiments. The bioreactor contained 50 mL of MOPS media, as well as a 0.5^″^ magnetic stir bar to facilitate mixing. The bioreactor was suspended in a water bath, maintained at 37°C, using a magnetic stirrer hot plate (Fisher Scientific). PAPA NONOate was delivered using a 2 channel, 8 roller, Ismatec REGLO ICC Digital Peristaltic Pump (Cole Palmer). 30-gauge, regular bevel, stainless steel needles (Covidien) were fastened into the ends of the tubing to create a drip system to facilitate delivery into the bioreactor. Delivery schedules were programmed using the associated software on a Dell Latitude E7440 with an Intel Core i5 CPU processor at 2.50 GHz. One channel was programmed to deliver NONOate and the second channel was programmed to deliver 10 mM NaOH, to maintain a constant volume delivered per unit time across delivery schema (5 mL/h). Prior to delivery, each channel was run for 2 min at a flow rate of 50 μL/min, to ensure that tubing had been primed and loaded with their respective solutions (approximately 8 equivalent volumes of fluid through the tubing). We note that experiments on this system were performed for up to a few hours. When longer time periods were assessed (e.g., 24 h), considerable volume loss due to evaporation from bioreactor was observed (50% or more), and delivery of 10 mM NaOH solutions over those time periods resulted in much higher media pH levels (e.g., above 9). These constraints limited experiments that were performed to several hours.

### [NO] and [O_2_] Measurements

NO concentrations were measured continuously using a 2 mm NO sensing probe (WPI). The probe was calibrated daily using the manufacturer’s instructions. Briefly, this was accomplished by delivering increasing doses of *S*-nitroso-*N*-acetyl-d,l-penicillamine (SNAP) (Cayman Chemical) to a 10 mM copper chloride (II) solution. A proportionality factor of 0.457 molecules of NO per molecule of SNAP (Chou and Brynildsen, 2019) was used to convert the raw signal generated (pico Amps) to units of NO concentration (μM). For NO assays where pico Amp measurements following clearance fell slightly below baseline, [NO] data were set to zero.

O_2_ concentration present in the bioreactor was continuously monitored using OXROB10 robust O_2_ probe (Pyroscience) attached to a FireStingO_2_ fiber-optic O_2_ meter (Pyroscience). Temperature was continuously monitored using TDIP15 temperature sensor (Pyroscience) and the probe signal automatically compensated for temperature fluctuations. The probe was calibrated daily using the manufacturer’s instructions.

### Absorbance Measurements (OD_600_, NO_2_^–^ and NO_3_^–^)

Cell density was measured during experiments by sampling 300 μL of solution from bioreactors and measuring absorbance at 600 nm using a microplate reader.

NO_2_^–^ and NO_3_^–^ concentrations were measured using a Nitrate/Nitrite Colorimetric Assay Kit (Cayman). Samples consisted of biological triplicates that were each measured in technical triplicates. The NO_2_^–^ concentration in samples was estimated by adding Griess reagents to samples, which converted them to Azo products. Following this, absorbance was measured at 540 nm using a microplate reader. A calibration curve was constructed using various concentrations of an NO_2_^–^ standard solution. A similar process was used to measure total NO_2_^–^ and NO_3_^–^ concentration in samples. However, an additional step, involving the addition of nitrate reductase and cofactors, was used to convert NO_3_^–^ to NO_2_^–^. Similarly, a calibration curve was constructed using various concentrations of an NO_3_^–^ standard solution. NO_3_^–^ concentration was calculated by subtracting the NO_2_^–^ concentration that was measured from the combined NO_2_^–^ and NO_3_^–^ concentration measurement. For more details on the procedure, refer to the manufacturer’s instructions.

### NO Consumption Assays

*E. coli* were taken from a −80°C stock, inoculated into a test tube with 1 ml of LB broth and incubated for 4 h at 37°C and 250 rpm. Following this, 10 μL were extracted from the test tube and transferred to a second test tube containing 1 mL of MOPS minimal media. The second test tube was incubated for 16 h at 37°C and 250 rpm. After 16 h, the overnight culture was used to inoculate a 250 mL baffled flask with 20 mL MOPS media at an OD_600_ of 0.01. The flask culture was grown to mid-exponential phase (OD_600_ = 0.2) and transferred to a pre-warmed (37°C) 50 mL falcon tube. The falcon tube was centrifuged at 4000 rpm, for 10 min at 37°C. Following this, 16 mL of MOPS were removed from the falcon tube, carefully avoiding the pellet of cells. The pellet was re-suspended in the remaining 4 mL and 1 mL was transferred to four separate pre-warmed (37°C) microcentrifuge tubes. The tubes were then centrifuged at 15,000 rpm for 3 min. Nine hundred and eighty microliter of media was removed from each microcentrifuge tube and the cell pellets were resuspended in 1 mL of pre-warmed MOPS media. The resuspended culture was used to inoculate a bioreactor with 50 mL MOPS media at an OD_600_ of 0.05. Five minutes after inoculation, NONOate delivery was initiated, either as bolus or through a delivery scheme implemented using the digital peristaltic pump.

### Mathematical Modeling

#### Model Construction

The model was constructed and used in previous studies (Robinson and Brynildsen, 2013, 2015, 2016b; Robinson et al., 2014b). In brief, the mathematical model is a system of ordinary differential equations that describes the change in concentration of numerous biochemical species, upon exposure to NO, within the cell as well as the extracellular environment, as a function of reaction rates and stoichiometric coefficients.

(2) $$\frac{d\,\vec{C}}{d\,t}=\hat{\text{S}}\cdot {\vec{r}}_{I}-\text{d}\cdot \vec{C}$$

Where $\vec{C}$ represents a vector of species concentrations. $\hat{\text{S}}$ is a scaled reaction stoichiometry matrix and ${\vec{r}}_{I}$ is a vector of intensive reaction rates, which itself is a function of species concentrations and kinetic parameters. **d** represents a diagonal matrix of species-specific dilution terms as a result of volume expansion during NONOate delivery and cellular growth. The model was partitioned into extracellular and intracellular compartments, assuming rapid diffusion of NO and O_2_ across the cell membrane. This was done to facilitate parameter optimization and model validation. Initial species concentrations, reaction rates and reaction structures were derived from the literature or trained on experimental data. MATLAB 2017b was used to run all simulations. For more information, on model construction and the specific reactions and species relevant to the model, refer to (Supplementary Methods and Supplementary Tables S1–S3).

#### Incorporation of NONOate Delivery Module

Delivery was incorporated into the differential equation for [NONOate] by including a delivery function, capable of taking one of four functional forms.

(3) $$N_{N\,O\,N\,O\,a\,t\,e_{|t=0}}+\int_{0}^{t_{f}}f_{d\,e\,l}\,dt=\omega$$

Where *N*_*NONOate_—t=0*_ represents the number of moles of NONOate introduced as a bolus at the onset of delivery. *f*_*del*_ is the NONOate delivery function (μmol/h); *t*_*f*_ represents the duration of delivery (h); ω represents the total payload delivered (μmol). For more details, refer to (Supplementary Methods).

#### Incorporation of Bacterial Growth

Bacterial growth was modeled as a function of cell density:

(4) $$\frac{d\,X}{d\,t}=\mu \cdot X$$

Where μ represents the specific growth rate and *X* represents cell density. *X* was assumed to vary linearly with optical density at 600 nm (*OD*_*600*_), such that *k*⋅*X* = *OD*_600_ (Myers et al., 2013). μ was modeled as a 1st order Hill-type equation that depended on the concentrations of available cytochromes *bo* and *bd*:

(5) $$\mu =\mu_{m\,a\,x}\cdot \frac{\left[C\,y\,t\,o\,c\,h\,r\,o\,m\,e_{b\,o}\right]+\left[C\,y\,t\,o\,c\,h\,r\,o\,m\,e_{b\,d}\right]}{K_{\mu }+\left[C\,y\,t\,o\,c\,h\,r\,o\,m\,e_{b\,o}\right]+\left[C\,y\,t\,o\,c\,h\,r\,o\,m\,e_{b\,d}\right]}$$

#### Incorporation of Growth-Dependent Translation Rate

Previously, we had chosen to model the rate of protein production as a function of mRNA transcripts with the inclusion of an [O_2_] dependency, such that increased [O_2_] led to increased translation rate (Robinson and Brynildsen, 2016b). The inspiration for this was that cells grew faster at higher O_2_ tensions, and translation is known to vary closely with specific growth rate (Neidhardt and Magasanik, 1960; Roller et al., 2016; Dai et al., 2017; Zhu and Dai, 2018).

(6) $$\frac{d\,\left[P\,r\,o\,t\,e\,i\,n\right]}{d\,t}=k_{t\,r\,a\,n\,s\,l\,a\,t\,e}\cdot \left[m\,R\,N\,A\right]\cdot \left(1+k_{a\,c\,t,O_{2}}\cdot \frac{\left[O_{2}\right]}{K_{O_{2}}+\left[O_{2}\right]}\right)-k_{d\,e\,g}\cdot \left[P\,r\,o\,t\,e\,i\,n\right]$$

Where “Protein” represents either Hmp, NorV, or NrfA. mRNA represents the associated mRNA for each protein (mRNA_*H*__*mp*_, mRNA_*N*__*or*__*V*_, mRNA_*N*__*rf*__*A*_). However, with the addition of growth to the model, we replaced the [O_2_] dependency term with a growth dependency term, which more explicitly exemplifies the connection between specific growth rate and translation rate.

(7) $$\frac{d\,\left[P\,r\,o\,t\,e\,i\,n\right]}{d\,t}=k_{t\,r\,a\,n\,s\,l\,a\,t\,e}\cdot \left[m\,R\,N\,A\right]\cdot \left(1+k_{g\,r\,o\,w\,t\,h}\cdot \frac{\mu }{\mu_{m\,a\,x}}\right)-k_{d\,e\,g}\cdot \left[P\,r\,o\,t\,e\,i\,n\right]$$

Where protein production is modeled with a growth dependency term as opposed to an O_2_ dependency term. Substituting Equation (5) into Equation (7), protein production can be re-written as a function of terminal cytochrome oxidases.

(8) $$\frac{d\,\left[P\,r\,o\,t\,e\,i\,n\right]}{d\,t}=k_{t\,r\,a\,n\,s\,l\,a\,t\,e}\cdot \left[mRNA\right]\cdot (1+k_{g\,r\,o\,w\,t\,h}\cdot \frac{\left[C\,y\,t\,o\,c\,h\,r\,o\,m\,e_{b\,o}\right]+\left[C\,y\,t\,o\,c\,h\,r\,o\,m\,e_{b\,d}\right]}{K_{\mu }+\left[C\,y\,t\,o\,c\,h\,r\,o\,m\,e_{b\,o}\right]+\left[C\,y\,t\,o\,c\,h\,r\,o\,m\,e_{b\,d}\right]})-k_{d\,e\,g}\cdot \left[P\,r\,o\,t\,e\,i\,n\right]$$

Where protein production in Equation (8) is a function of terminal cytochrome oxidases *bo* and *bd*, as opposed to a function of [O_2_]. This modified form represents a direct relationship between translation and cellular respiration, where the larger the concentration of uninhibited cytochromes, the greater the rates of respiration, which leads to faster cellular growth rates and accelerated rates of protein production.

#### Incorporation of Extracellular and Intracellular Dilution

Previously, the model assumed a fixed volume in the bioreactor during the course of experiments, and that changes in concentration of individual species were only a result of consumption and production. However, with the implementation of the low flow drip system, the volume of the bioreactor continuously changed during delivery. As a result, species relevant to the extracellular environment were continuously diluted. In a similar vein, with the addition of growth, we could no longer assume a fixed cellular volume as a function of time. As cells grow, so does the cellular volume in the reactor, and species relevant to the intracellular environment are diluted in growing cells. Therefore, in the rate equation for each species, we incorporated a term to account for dilution:

(9) $$d=\frac{\frac{d\,V_{i}}{d\,t}}{V_{i}}$$

Where *V*_*i*_ represents the volume compartment in which the species exists, where *i* can be extracellular, intracellular, or total. For more details, regarding model compartmentalization and derivation of the dilution term refer to (Supplementary Methods).

### Parameter Optimization

Uncertain model parameters were fitted to experimental data, using the MATLAB function *lsqcurvefit*. Specifically, the algorithm involves a non-linear least squares optimization algorithm that searched for optimal parameter sets by minimizing the variance weighted sum of squared residuals between experimental NO (and/or O_2_) curves and model simulations. Due to the compartmentalization of the model, different sets of unknown parameters were estimated independently using specific experimental conditions. The uncertain parameters fall into three categories: parameters relevant to NO reactions in the extracellular environment, parameters relevant to growth and cellular respiration, and parameters relevant to NO reactions in the cellular environment.

#### Estimating Extracellular Parameters

The product of the O_2_ mass transfer coefficient and surface area to volume ratio of the media in the bioreactor ($k_{L_{O_{2}}}\cdot \frac{A}{V}$) was estimated from O_2_ curves generated after purging O_2_ with N_2_ gas from bioreactors containing 50 and 55 mL MOPS media (Supplementary Figure S8). The rate of autoxidation, PAPA NONOate degradation rate, and rate of NO loss to the gas phase were trained simultaneously on both [NO] and [O_2_] curves generated from bolus delivery of 6 and 18 μmol of PAPA NONOate in cell-free media (Supplementary Figure S9). For more information on the parameters included in the optimization, refer to (Supplementary Methods and Supplementary Table S4).

#### Estimating O_2_ Respiratory Parameters

Parameters relevant to cellular respiration, namely cellular growth, ubiquinol-oxygen oxidoreduction, and ubiquinone reduction (16 parameters in total), were trained on [O_2_] and OD_600_ data generated from monitoring cells seeded at an OD_600_ of 0.025 and treated with 0, 50 and 1000 μM KCN (Supplementary Figure S2), which inhibits respiration and halt growth. For more information on the parameters included in the optimization, refer to (Supplementary Table S5).

#### Estimating NO Cellular Parameters

Uncertain cellular parameters related to NO detoxification (21 parameters in total) were optimized on 8 sets of [NO] and [O_2_] data obtained for bolus, constant, ramp down, and ramp up delivery regimens at 6 and 18 μmol. We note that a variety of training protocols could have been employed. We elected to use all four delivery dynamics at both 8 and 16 μmol for training because of the distinct outcomes observed in different conditions (e.g., immediate cessation of growth, lack of cessation of growth, delayed cessation of growth). We considered this diversity to be important for the ability of the model to extrapolate to conditions that it was not trained on, such as the 24 μmol dataset. For more information on the parameters included in the optimization, refer to (Supplementary Table S6).

#### Model Discrimination

Parameter sets were compared using Evidence Ratios (ER), which represent the likelihood of a given parameter set relative to the best set identified. Parameter sets with ER > 10, representing a less than 10% likelihood, were discarded. All parameters sets with ER < 10 were retained and used as initial points for an out-of-equilibrium adaptive Metropolis Markov Chain Monte Carlo (MCMC) process to further explore parameter space. If the MCMC algorithm generated a parameter set such that the initial point had an ER > 10 relative to the new minimum, the process was repeated using the best parameter set obtained from MCMC as the initial point. For more information on the model selection process, refer to (Robinson and Brynildsen, 2016b).

#### Algorithm to Identify Composite Dosing Schedules

For specified NO concentrations above 1 μM, the following algorithm was applied to identify dosing schema to maintain steady state levels, subject to the constraint of a total 24 μmol payload. Initial boluses (α) were determined such that simulated [NO] peaks equaled desired steady state concentrations. The vectors of species concentrations at the time of those peaks were used as initial conditions for secondary simulations where at each time step the concentrations of NONOate were solved to maintain d[NO]/dt = 0. The output of this simulation was a vector of NONOate concentrations at each time point, which was used to compute vectors of values corresponding to d[NONOate]/dt, and the rates of loss of NONOate (through decay and dilution) at each time point. These three vectors were added together, which yielded a vector corresponding to the NONOate delivery rate (*f*_*del*_) required to maintain steady state.

(10) $$f_{d\,e\,l}=\frac{d\,\left[N\,O\,N\,O\,a\,t\,e\right]}{d\,t}+k_{d\,e\,g}\cdot \left[N\,O\,N\,O\,a\,t\,e\right]+d_{N\,O\,N\,O\,a\,t\,e}$$

Lastly, *f*_*del*_ was truncated at t = T, such that $\int_{0}^{T}f_{d\,e\,l}\,dt=24-\alpha$.

In addition to determining dosing schedules to maintain [NO] at specified steady state levels, we attempted to enhance the antibacterial activity of NO by formulating the task as an optimization problem. Specifically, we attempted to maximize the amount of time [NO] was at or above the threshold to inhibit 99% of cytochromes (∼1.2 μM) by using the MATLAB optimization function *fmincon*. For this purpose, we considered dosing schedules that could be captured by third order polynomials. Parameters that were allowed to vary during the optimization were the coefficients of the polynomial and the total delivery time, and solutions were similarly constrained to cumulative payloads of 24 μmol. We tried two different approaches for initialization. In the first, the polynomial coefficients and delivery time were obtained from a least-squares fitting to the 2.2 μM steady state dosing scheme (best predicted scheme from steady state approach). The optimization algorithm then used those values and α from the steady state solution as a jumping off point to maximize the amount of time at or above ∼1.2 μM NO. This enabled a focused search around the best steady state solution for better performing schedules. In the second initialization approach, we used 100 sets of randomly selected polynomial coefficients and values of α subject to a total payload of 24 μmol, which effectively set the total delivery time for each initialization. The optimization algorithm then used those values as initial points to maximize the amount of time at or above ∼1.2 μM NO. This allowed a broader swath of parameter space to be searched for solutions better than the best steady state solution. For both types of initialization, the optimization procedure did not yield solutions that were better than the best dosing schedule from the steady state approach. This could have occurred due to the algorithm getting trapped in local optima or third order polynomials giving insufficient flexibility to reach globally optimal solutions. Given the depth and breadth of potential algorithms and optimization formulations there remains the possibility that better solutions than that provided by the steady state approach could be found in future studies.

## Data Availability Statement

The datasets generated for this study are available on request to the corresponding author.

## Author Contributions

Experiments and analyses were designed by DS and MB. DS performed the experiments and analyzed the data. The manuscript was written by DS and MB.

## Conflict of Interest

The authors declare that the research was conducted in the absence of any commercial or financial relationships that could be construed as a potential conflict of interest.

## References

[B1] Bang I.-S. Liu L. Vazquez-Torres A. Crouch M.-L. Stamler J. S. Fang F. C. (2006). Maintenance of nitric oxide and redox homeostasis by the *Salmonella* flavohemoglobin hmp. *J. Biol. Chem.* 281 28039–28047. 10.1074/jbc.M605174200 16873371

[B2] Baron S. (ed.) (1996). “Chapter 4: Bacterial metabolism,” in *Medical Microbiology* (Galveston, TX: University of Texas Medical Branch at Galveston). 21413252

[B3] Bowman L. A. H. McLean S. Poole R. K. Fukuto J. M. (2011). The diversity of microbial responses to nitric oxide and agents of nitrosative stress. *Adv. Microb. Physiol.* 59 135–219. 10.1016/B978-0-12-387661-4.00006-9 22114842

[B4] Brynildsen M. P. Winkler J. A. Spina C. S. MacDonald I. C. Collins J. J. (2013). Potentiating antibacterial activity by predictably enhancing endogenous microbial ROS production. *Nat. Biotechnol.* 31 160–165. 10.1038/nbt.2458 23292609 PMC3568245

[B5] Choi H. W. Kim J. Kim J. Kim Y. Song H. B. Kim J. H. (2016). light-induced acid generation on a gatekeeper for smart nitric oxide delivery. *ACS Nano* 10 4199–4208. 10.1021/acsnano.5b07483 26953516

[B6] Chou W. K. Brynildsen M. P. (2019). Loss of DksA leads to multi-faceted impairment of nitric oxide detoxification by *Escherichia coli*. *Free Radic. Biol. Med.* 130 288–296. 10.1016/j.freeradbiomed.2018.10.435 30366060

[B7] Corker H. Poole R. K. (2003). Nitric oxide formation by *Escherichia coli*: dependence on nitrite reductase, the no-sensing regulator Fnr, and flavohemoglobin hmp. *J. Biol. Chem.* 278 31584–31592. 10.1074/jbc.M303282200 12783887

[B8] Dai X. Zhu M. Warren M. Balakrishnan R. Patsalo V. Okano H. (2017). Reduction of translating ribosomes enables *Escherichia coli* to maintain elongation rates during slow growth. *Nat. Microbiol.* 2:16231 10.1038/nmicrobiol.2016.231 PMC5346290 27941827

[B9] Evans M. A. Huang P.-J. Iwamoto Y. Ibsen K. N. Chan E. M. Hitomi Y. (2018). Macrophage-mediated delivery of light activated nitric oxide prodrugs with spatial, temporal and concentration control. *Chem. Sci.* 9 3729–3741. 10.1039/C8SC00015H 29780505 PMC5939611

[B10] Fang F. C. (2004). Antimicrobial reactive oxygen and nitrogen species: concepts and controversies. *Nat. Rev. Microbiol.* 2 820–832. 10.1038/nrmicro1004 15378046

[B11] Flannagan R. S. Cosío G. Grinstein S. (2009). Antimicrobial mechanisms of phagocytes and bacterial evasion strategies. *Nat. Rev. Microbiol.* 7 355–366. 10.1038/nrmicro2128 19369951

[B12] Friedman A. Blecher K. Sanchez D. Tuckman-Vernon C. Gialanella P. Friedman J. M. (2011). Susceptibility of Gram-positive and -negative bacteria to novel nitric oxide-releasing nanoparticle technology. *Virulence* 2 217–221. 10.4161/viru.2.3.16161 21577055

[B13] Gardner A. M. Gardner P. R. (2002). Flavohemoglobin detoxifies nitric oxide in aerobic, but not anaerobic, *Escherichia coli*: evidence for a novel inducible anaerobic nitric oxide-scavenging activity. *J. Biol. Chem.* 277 8166–8171. 10.1074/jbc.M110470200 11751864

[B14] Gardner P. (2005). Nitric oxide dioxygenase function and mechanism of flavohemoglobin, hemoglobin, myoglobin and their associated reductases. *J. Inorg. Biochem.* 99 247–266. 10.1016/j.jinorgbio.2004.10.003 15598505

[B15] Gardner P. R. (2012). Hemoglobin: a nitric-oxide dioxygenase. *Scientifica* 2012 1–34. 10.6064/2012/683729 PMC3820574 24278729

[B16] Haas A. (2007). The phagosome: compartment with a license to kill. *Traffic* 8 311–330. 10.1111/j.1600-0854.2006.00531.x 17274798

[B17] Hasanzadeh Kafshgari M. Delalat B. Harding F. J. Cavallaro A. Mäkilä E. Salonen J. (2016). Antibacterial properties of nitric oxide-releasing porous silicon nanoparticles. *J. Mater. Chem. B* 4 2051–2058. 10.1039/C5TB02551F 32263082

[B18] Heilman B. Mascharak P. K. (2013). Light-triggered nitric oxide delivery to malignant sites and infection. *Philos. Trans. R. Soc. Math. Phys. Eng. Sci.* 371:20120368 10.1098/rsta.2012.0368 23776301

[B19] Henard C. A. Vázquez-Torres A. (2011). Nitric oxide and *Salmonella* pathogenesis. *Front. Microbiol.* 2:84 10.3389/fmicb.2011.00084 PMC3153045 21833325

[B20] Ho K. K. K. Ozcelik B. Willcox M. D. P. Thissen H. Kumar N. (2017). Facile solvent-free fabrication of nitric oxide (NO)-releasing coatings for prevention of biofilm formation. *Chem. Commun.* 53 6488–6491. 10.1039/C7CC02772A 28569892

[B21] Hobbs M. R. Udhayakumar V. Levesque M. C. Booth J. Roberts J. M. Tkachuk A. N. (2002). A new NOS2 promoter polymorphism associated with increased nitric oxide production and protection from severe malaria in Tanzanian and Kenyan children. *Lancet* 360:8. 10.1016/S0140-6736(02)11474-7 12433515

[B22] Hogg N. Kalyanaraman B. (1999). Nitric oxide and lipid peroxidation. *Biochim. Biophys. Acta BBA Bioenerg.* 1411 378–384. 10.1016/S0005-2728(99)00027-24 10320670

[B23] Hou J. Pan Y. Zhu D. Fan Y. Feng G. Wei Y. (2019). Targeted delivery of nitric oxide via a ‘bump-and-hole’-based enzyme–prodrug pair. *Nat. Chem. Biol.* 15 151–160. 10.1038/s41589-018-0190-195 30598545

[B24] Hurford W. E. (2005). Nitric oxide as a bactericidal agent: is the cure worse than the disease? *Respir. Care* 50 1428–1429. 16253148

[B25] Jones M. L. Ganopolsky J. G. Labbé A. Prakash S. (2010). A novel nitric oxide producing probiotic patch and its antimicrobial efficacy: preparation and in vitro analysis. *Appl. Microbiol. Biotechnol.* 87 509–516. 10.1007/s00253-010-2490-x 20300748

[B26] Kim J. Saravanakumar G. Choi H. W. Park D. Kim W. J. (2014). A platform for nitric oxide delivery. *J. Mater. Chem. B* 2 341–356. 10.1039/C3TB21259A 32261379

[B27] Kun J. F. Mordmüller B. Perkins D. J. May J. Mercereau-Puijalon O. Alpers M. (2001). Nitric oxide synthase 2 lambaréné (G-954C), increased nitric oxide production, and protection against malaria. *J. Infect. Dis.* 184 330–336. 10.1086/322037 11443559

[B28] Liang H. Nacharaju P. Friedman A. Friedman J. M. (2015). Nitric oxide generating/releasing materials. *Future Sci. OA* 1:FSO54 10.4155/fso.15.54 PMC4739797 26855790

[B29] Lu Y. Slomberg D. L. Schoenfisch M. H. (2014). Nitric oxide-releasing chitosan oligosaccharides as antibacterial agents. *Biomaterials* 35 1716–1724. 10.1016/j.biomaterials.2013.11.015 24268196 PMC3889664

[B30] MacMicking J. D. Nathan C. Hom G. Chartrain N. Fletcher D. S. Trumbauer M. (1995). Altered responses to bacterial infection and endotoxic shock in mice lacking inducible nitric oxide synthase. *Cell* 81 641–650. 10.1016/0092-8674(95)90085-90083 7538909

[B31] Martinez L. R. Han G. Chacko M. Mihu M. R. Jacobson M. Gialanella P. (2009). Antimicrobial and healing efficacy of sustained release nitric oxide nanoparticles against staphylococcus aureus skin infection. *J. Invest. Dermatol.* 129 2463–2469. 10.1038/jid.2009.95 19387479

[B32] Myers J. A. Curtis B. S. Curtis W. R. (2013). Improving accuracy of cell and chromophore concentration measurements using optical density. *BMC Biophys.* 6:4 10.1186/2046-1682-6-4 PMC3663833 24499615

[B33] Nablo B. J. Rothrock A. R. Schoenfisch M. H. (2005). Nitric oxide-releasing sol–gels as antibacterial coatings for orthopedic implants. *Biomaterials* 26 917–924. 10.1016/j.biomaterials.2004.03.031 15353203

[B34] Neidhardt F. C. Magasanik B. (1960). Studies on the role of ribonucleic acid in the growth of bacteria. *Biochim. Biophys. Acta* 42 99–116. 10.1016/0006-3002(60)90757-90755 13728193

[B35] O’Donnell V. B. Freeman B. A. (2001). Interactions between nitric oxide and lipid oxidation pathways: implications for vascular disease. *Circ. Res.* 88 12–21. 10.1161/01.RES.88.1.12 11139468

[B36] Pfeiffer S. Lass A. Schmidt K. Mayer B. (2001). Protein tyrosine nitration in cytokine-activated murine macrophages: involvement of a peroxidase/nitrite pathway rather than peroxynitrite. *J. Biol. Chem.* 276 34051–34058. 10.1074/jbc.M100585200 11425852

[B37] Poole R. K. (2005). Nitric oxide and nitrosative stress tolerance in bacteria. *Biochem. Soc. Trans.* 33 176–180. 10.1042/BST0330176 15667299

[B38] Poole R. K. Hughes M. N. (2000). New functions for the ancient globin family: bacterial responses to nitric oxide and nitrosative stress. *MicroReview. Mol. Microbiol.* 36 775–783. 10.1046/j.1365-2958.2000.01889.x 10844666

[B39] Quinn J. F. Whittaker M. R. Davis T. P. (2015). Delivering nitric oxide with nanoparticles. *J. Control. Rel.* 205 190–205. 10.1016/j.jconrel.2015.02.007 25665865

[B40] Radi R. (2018). Oxygen radicals, nitric oxide, and peroxynitrite: redox pathways in molecular medicine. *Proc. Natl. Acad. Sci. U.S.A.* 115 5839–5848. 10.1073/pnas.1804932115 29802228 PMC6003358

[B41] Radtke A. L. O’Riordan M. X. D. (2006). Intracellular innate resistance to bacterial pathogens. *Cell. Microbiol.* 8 1720–1729. 10.1111/j.1462-5822.2006.00795.x 16939532

[B42] Reichner J. S. Meszaros A. J. Louis C. A. Henry W. L. Mastrofrancesco B. Martin B.-A. (1999). Molecular and metabolic evidence for the restricted expression of inducible nitric oxide synthase in healing wounds. *Am. J. Pathol.* 154 1097–1104. 10.1016/S0002-9440(10)65362-X 10233848 PMC1866555

[B43] Riccio D. A. Schoenfisch M. H. (2012). Nitric oxide release: Part I. *Macromolecular scaffolds*. *Chem. Soc. Rev.* 41:3731 10.1039/c2cs15272j PMC3341515 22362355

[B44] Robinson J. Brynildsen M. (2016a). Construction and experimental validation of a quantitative kinetic model of nitric oxide stress in enterohemorrhagic *Escherichia coli* O157:H7. *Bioengineering* 3:9 10.3390/bioengineering3010009 PMC5597167 28952571

[B45] Robinson J. L. Adolfsen K. J. Brynildsen M. P. (2014a). Deciphering nitric oxide stress in bacteria with quantitative modeling. *Curr. Opin. Microbiol.* 19 16–24. 10.1016/j.mib.2014.05.018 24983704 PMC4130159

[B46] Robinson J. L. Miller R. V. Brynildsen M. P. (2014b). Model-driven identification of dosing regimens that maximize the antimicrobial activity of nitric oxide. *Metab. Eng. Commun.* 1 12–18. 10.1016/j.meteno.2014.08.001 PMC8193240 34150500

[B47] Robinson J. L. Brynildsen M. P. (2013). A kinetic platform to determine the fate of nitric oxide in *Escherichia coli*. *PLoS Comput. Biol.* 9:e1003049 10.1371/journal.pcbi.1003049 PMC3642044 23658508

[B48] Robinson J. L. Brynildsen M. P. (2015). An ensemble-guided approach identifies ClpP as a major regulator of transcript levels in nitric oxide-stressed *Escherichia coli*. *Metab. Eng.* 31 22–34. 10.1016/j.ymben.2015.06.005 26112956

[B49] Robinson J. L. Brynildsen M. P. (2016b). Discovery and dissection of metabolic oscillations in the microaerobic nitric oxide response network of *Escherichia coli*. *Proc. Natl. Acad. Sci. U.S.A.* 113 E1757–E1766. 10.1073/pnas.1521354113 26951670 PMC4812703

[B50] Roller B. R. K. Stoddard S. F. Schmidt T. M. (2016). Exploiting rRNA operon copy number to investigate bacterial reproductive strategies. *Nat. Microbiol.* 1:16160 10.1038/nmicrobiol.2016.160 PMC5061577 27617693

[B51] Roveda A. C. Papa T. B. R. Castellano E. E. Franco D. W. (2014). PAMAM dendrimers functionalized with ruthenium nitrosyl as nitric oxide carriers. *Inorganica Chim. Acta* 409 147–155. 10.1016/j.ica.2013.07.009

[B52] Sacco S. A. Adolfsen K. J. Brynildsen M. P. (2017). An integrated network analysis identifies how ArcAB enables metabolic oscillations in the nitric oxide detoxification network of *Escherichia coli*. *Biotechnol. J.* 12:1600570 10.1002/biot.201600570 28449226

[B53] Sadrearhami Z. Nguyen T.-K. Namivandi-Zangeneh R. Jung K. Wong E. H. H. Boyer C. (2018). Recent advances in nitric oxide delivery for antimicrobial applications using polymer-based systems. *J. Mater. Chem. B* 6 2945–2959. 10.1039/C8TB00299A 32254331

[B54] Schairer D. O. Chouake J. S. Nosanchuk J. D. Friedman A. J. (2012a). The potential of nitric oxide releasing therapies as antimicrobial agents. *Virulence* 3 271–279. 10.4161/viru.20328 22546899 PMC3442839

[B55] Schairer D. O. Martinez L. R. Blecher K. Chouake J. S. Nacharaju P. Gialanella P. (2012b). Nitric oxide nanoparticles: pre-clinical utility as a therapeutic for intramuscular abscesses. *Virulence* 3 62–67. 10.4161/viru.3.1.18816 22286699 PMC3337151

[B56] Seabra A. B. Durán N. (2010). Nitric oxide-releasing vehicles for biomedical applications. *J. Mater. Chem.* 20 1624–1637. 10.1039/B912493B

[B57] Soria S. de Anda R. Flores N. Romero-Garcia S. Gosset G. Bolívar F. (2013). New insights on transcriptional responses of genes involved in carbon central metabolism, respiration and fermentation to low ATP levels in *Escherichia coli*: new insights on transcriptional responses of genes in *E. coli*. *J. Basic Microbiol.* 53 365–380. 10.1002/jobm.201100525 22914992

[B58] Sortino S. (2010). Light-controlled nitric oxide delivering molecular assemblies. *Chem. Soc. Rev.* 39:2903 10.1039/b908663n 20556272

[B59] Spiro S. Zhang M. Q. Mehta H. H. Liu Y. (2015). Genome-wide analysis of the response to nitric oxide in uropathogenic *Escherichia coli* CFT073. *Microb. Genomics* 1:e000031 10.1099/mgen.0.000031 PMC5320621 28348816

[B60] Stern A. M. Zhu J. (2014). An introduction to nitric oxide sensing and response in bacteria. *Adv. Appl. Microbiol.* 87 187–220. 10.1016/B978-0-12-800261-2.00005-0 24581392

[B61] Stevanin T. M. Poole R. K. Demoncheaux E. A. G. Read R. C. (2002). Flavohemoglobin Hmp protects *Salmonella enterica* Serovar *Typhimurium* from nitric oxide-related killing by human macrophages. *Infect. Immun.* 70 4399–4405. 10.1128/IAI.70.8.4399-4405.2002 12117950 PMC128135

[B62] Sun B. Slomberg D. L. Chudasama S. L. Lu Y. Schoenfisch M. H. (2012). Nitric oxide-releasing dendrimers as antibacterial agents. *Biomacromolecules* 13 3343–3354. 10.1021/bm301109c 23013537 PMC3482834

[B63] Svensson L. Poljakovic M. Säve S. Gilberthorpe N. Schön T. Strid S. (2010). Role of flavohemoglobin in combating nitrosative stress in uropathogenic *Escherichia coli* – Implications for urinary tract infection. *Microb. Pathog.* 49 59–66. 10.1016/j.micpath.2010.04.001 20399845

[B64] Tfouni E. Truzzi D. R. Tavares A. Gomes A. J. Figueiredo L. E. Franco D. W. (2012). Biological activity of ruthenium nitrosyl complexes. *Nitric Oxide* 26 38–53. 10.1016/j.niox.2011.11.005 22178685

[B65] Thomas D. D. Ridnour L. A. Isenberg J. S. Flores-Santana W. Switzer C. H. Donzelli S. (2008). The chemical biology of nitric oxide: implications in cellular signaling. *Free Radic. Biol. Med.* 45 18–31. 10.1016/j.freeradbiomed.2008.03.020 18439435 PMC2572721

[B66] Toledo J. C. Augusto O. (2012). Connecting the chemical and biological properties of nitric oxide. *Chem. Res. Toxicol.* 25 975–989. 10.1021/tx300042g 22449080

[B67] Trotter E. W. Rolfe M. D. Hounslow A. M. Craven C. J. Williamson M. P. Sanguinetti G. (2011). Reprogramming of *Escherichia coli* K-12 metabolism during the initial phase of transition from an anaerobic to a micro-aerobic environment. *PLoS One* 6:e25501 10.1371/journal.pone.0025501 PMC3181329 21980479

[B68] Vázquez-Torres A. Fang F. C. (2005). Nitric oxide in *Salmonella* and *Escherichia coli* infections. *Ecosal Plus* 1:84 10.1128/ecosalplus.8.8.8 26443513

[B69] Vazquez-Torres A. Jones-Carson J. Mastroeni P. Ischiropoulos H. Fang F. C. (2000). Antimicrobial actions of the nadph phagocyte oxidase and inducible nitric oxide synthase in experimental Salmonellosis. I. effects on microbial killing by activated peritoneal macrophages in vitro. *J. Exp. Med.* 192 227–236. 10.1084/jem.192.2.227 10899909 PMC2193262

[B70] Wink D. A. Mitchell J. B. (1998). Chemical biology of nitric oxide: insights into regulatory, cytotoxic, and cytoprotective mechanisms of nitric oxide. *Free Radic. Biol. Med.* 25 434–456. 10.1016/S0891-5849(98)00092-6 9741580

[B71] Xiang H.-J. Guo M. Liu J.-G. (2017). Transition-metal nitrosyls for photocontrolled nitric oxide delivery: transition-metal nitrosyls for photocontrolled nitric oxide delivery. *Eur. J. Inorg. Chem.* 2017 1586–1595. 10.1002/ejic.201601135

[B72] Yang Y. Qi P. K. Yang Z. L. Huang N. (2015). Nitric oxide based strategies for applications of biomedical devices. *Biosurf. Biotribol.* 1 177–201. 10.1016/j.bsbt.2015.08.003

[B73] Zhu M. Dai X. (2018). On the intrinsic constraint of bacterial growth rate: *M. tuberculosis*’s view of the protein translation capacity. *Crit. Rev. Microbiol.* 44 455–464. 10.1080/1040841X.2018.1425672 29334314

