# Supplementary material for: Quantitative Modeling Extends the Antibacterial Activity of Nitric Oxide
Source: Front Physiol. 2020 Apr 17;11:330. doi: 10.3389/fphys.2020.00330 (PMC7181900; doi:10.3389/fphys.2020.00330)
Supplement: Supplementary file 4 [file Data_Sheet_1.pdf]

Supplementary Material

1 Supplementary Methods

1.1 NONOate delivery function

Given a payload ω µmol and delivery period $[0,\ t_f]$,

$$\frac{dN_{NONOate}}{dt} = f_{del} + f_{deg} \tag{1}$$

$$f_{deg} = -k_{deg} \cdot N_{NONOate} \tag{2}$$

$$f_{del} = a \cdot t + b \quad s.t. \quad N_{NONOate_{|t=0}} + \int_0^{t_f} f_{del}\ dt \ = \omega \tag{3}$$

Where a and b are defined by the principle mode implemented,

$$\text{Bolus} \begin{cases} a_b = 0 \\ b_b = 0 \\ N_{NONOate_{|t=0}} = \omega \end{cases} \tag{4}$$

$$\text{Constant} \begin{cases} a_c = 0 \\ b_c = \dfrac{\omega}{t_f} \\ N_{NONOate_{|t=0}} = 0 \end{cases} \tag{5}$$

$$\text{Rampdown} \begin{cases} a_d = \dfrac{-2 \cdot \left(t_f \cdot b_d - \ \omega\right)}{t_f^2} \\ b_d = [NONOate]_{res} \cdot v_{max} \\ N_{NONOate_{|t=0}} = 0 \end{cases} \tag{6}$$

$$\text{Rampup}\begin{cases} a_u = \dfrac{2 \cdot (\omega - t_f \cdot b_u)}{t_f^2} \\ b_u = [NONOate]_{res} \cdot v_{min} \\ N_{NONOate_{|t=0}} = 0 \end{cases} \tag{7}$$

Where $N_{NONOate_{|t=0}}$ represents the number of moles of NONOate introduced as a bolus at the onset of delivery. v_{max} and v_{min} are the maximum and minimum feasible volumetric flow rates programmed by the low flow delivery apparatus (0.63 mL/hr and 4.37 mL/hr respectively). $[NONOate]_{res}$ is the NONOate reservoir concentration, which was set based on the payload ω.

ω	$[NONOate]_{res}$
$6\,\mu mol$	$2400\,\mu M$
$18\,\mu mol$	$7200\,\mu M$
$24\,\mu mol$	$9600\,\mu M$

1.2 Volume function

The total volumetric flow rate was defined as a function of individual peristaltic pump flow rates, where the sum of flow rates was fixed at 5 mL/hr during the delivery period $[0,\ t_f]$.

$$\frac{dV}{dt} = g_{NONOate}(t) + g_{balance}(t) = 5\,\frac{\text{ml}}{hr} \tag{8}$$

Where $g_{NONOate}(t)$ represents the flow rate function from the NONOate reservoir and $g_{balance}(t)$ represents the flow rate function from a secondary reservoir containing blank solvent to maintain a fixed total volumetric flow rate. Both $g_{NONOate}(t)$ and $g_{balance}(t)$ are defined by the payload, delivery time, and principle mode implemented (refer to section 1.1). By integrating equation 8:

$$V(t) = V_0 + 5\,\frac{\text{ml}}{hr} \cdot t \tag{9}$$

Where V_0 is the initial volume of the bioreactor at $t = 0$.

1.3 Model compartmentalization

The model was partitioned into separate intracellular and extracellular compartments:

$$\frac{d\vec{C}}{dt} = \hat{\mathbf{S}} \cdot \vec{r}_I - \mathbf{d} \cdot \vec{C} \tag{10}$$

Where $\vec{C}$ represents a vector of species concentrations, $\hat{\mathbf{S}}$ represents a scaled reaction stoichiometry matrix, r_I represents a vector of intensive reaction rates ($\frac{mol}{vol \cdot time}$), and $\mathbf{d}$ represents a diagonal matrix of species specific dilution terms. The model assumes that concentration is not conserved across compartments (except for species that can diffuse across the membrane such as O_2, NO, and CO_2). In order to derive equation 10 and explicitly show the changes incorporated, the mass balance is written on a per mole basis:

$$\frac{d\vec{N}}{dt} = \mathbf{S} \cdot \vec{r}_E \tag{11}$$

where $\vec{r}_E$ represents a vector of extensive reaction rates ($\frac{mol}{time}$), $\vec{N}$ represents a vector of moles of each species, and **S** is a reaction stoichiometry matrix. However, reaction rates are generally written in units of ($\frac{mol}{vol \cdot time}$) as such $\vec{r}_E$ was expressed in terms of $\vec{r}_I$:

$$\frac{d\vec{N}}{dt} = \mathbf{S} \cdot \mathbf{V_{rxn}} \cdot \vec{r}_I \tag{12}$$

where $\vec{r}_I$ represents a vector of intensive reaction rates, $\mathbf{V_{rxn}}$ represents a diagonal matrix with elements corresponding to the volume of the compartment in which the reaction occurs (V_{cell} for intracellular reactions, V_{media} for extracellular reactions, and V_{total} for reactions that occur in both compartments). To rewrite $\frac{d\vec{N}}{dt}$, as $\frac{d\vec{C}}{dt}$, we substituted (volume · concentration) for moles and applied the product rule:

$$\frac{d\vec{N}}{dt} = \frac{d\left(\mathbf{V_{spec}} \cdot \vec{C}\right)}{dt} = \frac{d\mathbf{V_{spec}}}{dt} \cdot \vec{C} + \mathbf{V_{spec}} \cdot \frac{dC}{dt} \tag{13}$$

where $\mathbf{V_{spec}}$ represents a diagonal matrix with elements corresponding to the volume of the compartment in which each species exists (V_{cell} for intracellular species, V_{media} for extracellular species, and V_{total} for species capable of diffusing across the membrane).

$$\frac{d\vec{C}}{dt} = \mathbf{V_{spec}^{-1}} \cdot \mathbf{S} \cdot \mathbf{V_{rxn}} \cdot \vec{r}_I - \mathbf{V_{spec}^{-1}} \cdot \frac{d\mathbf{V_{spec}}}{dt}\vec{C} \tag{14}$$

The right-hand side of the equation was multiplied by $\frac{V_{total}}{V_{total}}$, and rearranged, using the commutative property of scalar multiplication:

$$\frac{d\vec{C}}{dt} = V_{total} \cdot \mathbf{V_{spec}^{-1}} \cdot \mathbf{S} \cdot \frac{\mathbf{V_{rxn}}}{V_{total}} \cdot \vec{r}_I - \mathbf{V_{spec}^{-1}} \cdot \frac{d\mathbf{V_{spec}}}{dt} \cdot \vec{C} \tag{15}$$

$$\frac{d\vec{C}}{dt} = \mathbf{F_{spec}^{-1}} \cdot \mathbf{S} \cdot \mathbf{F_{rxn}} \cdot \vec{r}_I - \mathbf{V_{spec}^{-1}} \cdot \frac{d\mathbf{V_{spec}}}{dt} \cdot \vec{C} \tag{16}$$

where:

$$\mathbf{F_{spec}} = \frac{\mathbf{V_{spec}}}{V_{total}}, \quad \mathbf{F_{rxn}} = \frac{\mathbf{V_{rxn}}}{V_{total}}, \quad \hat{\mathbf{S}} = \mathbf{F_{spec}^{-1}} \cdot \mathbf{S} \cdot \mathbf{F_{rxn}} \quad and \quad \mathbf{d} = \mathbf{V_{spec}^{-1}} \cdot \frac{d\mathbf{V_{spec}}}{dt} \tag{17}$$

1.4 Estimation of oxygen mass transfer coefficient

To estimate the oxygen mass transfer coefficient, an O_2 mass balance was written:

$$\frac{dN_{O_2}}{dt} = k_{L_{O_2}} \cdot A \cdot \left(C_{O_2}^* - C_{O_2}\right) \tag{18}$$

Where $\frac{dN_{O_2}}{dt}$ represents the change in moles of oxygen in the liquid phase as a function of time ($\frac{mol}{time}$), $k_{L_{O_2}}$ represents the liquid phase mass transfer coefficient for oxygen, A represents the area of the gas liquid interface, C_{O_2} represents the concentration of O_2 in the liquid, and $C_{O_2}^*$ represents the concentration of O_2 in the liquid that would be in equilibrium with the oxygen present in the gas phase. Substituting, applying the product rule of differentiation, and rearranging:

$$\frac{d(C_{O_2} \cdot V)}{dt} = V \cdot \frac{dC_{O_2}}{dt} + C_{O_2} \cdot \frac{dV}{dt} = k_{L_{O_2}} \cdot A \cdot (C_{O_2}^* - C_{O_2}) \tag{19}$$

$$\frac{dC_{O_2}}{dt} = \frac{k_{L_{O_2}} \cdot A}{V} \cdot (C_{O_2}^* - C_{O_2}) - \frac{1}{V} \cdot \frac{dV}{dt} \cdot C_{O_2} \tag{20}$$

Where $\frac{dC_{O_2}}{dt}$ represents the change in oxygen concentration in the liquid phase as a function of time ($\frac{mol}{vol \cdot time}$) and V represents the liquid volume. The combined parameter ($k_{L_{O_2}} \cdot A$) was optimized on O_2 measurements taken in both 50 and 55 mL MOPS media bioreactors after purging O_2 from the reactors with N_2 gas (Fig S2). Due the fixed volume of the bioreactor during measurements, the dilution term in equation 20 is zero and the expression reduces to:

$$\frac{dC_{O_2}}{dt} = \frac{k_{L_{O_2}} \cdot A}{V} \cdot (C_{O_2}^* - C_{O_2}) \tag{21}$$

($k_{L_{O_2}} \cdot A$) was estimated to be approximately $0.105 \frac{L}{hr}$, which yields ($k_{L_{O_2}} \cdot \frac{A}{V}$) of $2.1\ hr^{-1}$ and $1.9\ hr^{-1}$ in 50 and 55 mL respectively. The carbon dioxide mass transfer coefficient ($k_{L_{CO_2}}$) was assumed to have a similar value to those obtained for oxygen. We justify this approximation based on two criteria; i) dimensional analysis of forced convection (bioreactor is rapidly stirred) suggests that the Sherwood number will be a function of the Reynolds and Schmidt numbers, and thus only the diffusion coefficients, which should be quite similar between these molecules given their values in water at 25°C [1], would vary between carbon dioxide and oxygen in these relations, and ii) carbon dioxide plays a negligible role in NO metabolism and as such the need for rigorous measurement of carbon dioxide mass transfer was not deemed to be critical.

1.5 Modeling Hmp expression and activity

Hmp catalytic activity is modeled as a sub-network of individual reactions (Table S1 reactions 106-134), which includes Hmp reduction, inhibition of Hmp by NO, oxygen binding of Hmp, NO oxidation to NO_3^- and nitroxyl anion formation. Hmp expression is modeled through transcription, translation, and associated degradation of mRNA and protein (Table S1 reactions 139-153;159; 192; 195). Reaction mechanisms, rate constants, and species were obtained from

the literature (Table S1, S2 and S3) or trained on experimental data with parameter ranges obtained from the literature (Table S6).

1.6 Hmp equilibrium assumption in the absence of NO

The model assumes that in the absence of NO, Hmp mRNA and protein concentrations are held at steady state (non-zero, constant values). Moreover, the initial concentrations of non-NO bound Hmp isoforms, $HMP_{Fe^{3+}}$, $HMP_{Fe^{3+}H_2}$,$HMP_{Fe^{2+}H}$ and $HMP_{Fe^{2+}HO_2}$, are assumed to be in equilibrium and non-zero before NO treatment.

During model training, the initial concentration of $HMP_{Fe^{3+}}$, as well as several parameters related to Hmp transcription, translation and catalytic activity were optimized (Table S6). From the steady state and equilibrium assumptions, initial concentrations of HMP_{mRNA}, $HMP_{Fe^{3+}H_2}$, $HMP_{Fe^{2+}H}$, $HMP_{Fe^{2+}HO_2}$ and the basal transcription rate ($k_{transcr,basal}$) were identified with the following equations:

$$[HMP_{mRNA}]_0 = \frac{(k_{NADH} \cdot [NADH]_0 + k_{NADPH} \cdot [NADPH]_0 + \ k_{deg} + d_{HMP_{Fe^{3+}}}) \cdot [HMP_{Fe^{3+}}]_0}{k_{translate} \cdot \left(1 + k_{growth}\right) \cdot \left(\frac{[CYT_{bo}]_0 + [CYT_{bd}]_0}{K_\mu + [CYT_{bo}]_0 + [CYT_{bd}]_0}\right)} \tag{21}$$

$$k_{transcr,basal} = (k_{deg_{mRNA}} + d_{HMP_{mRNA}}) \cdot [HMP_{mRNA}]_0 \tag{22}$$

$$\left[HMP_{Fe^{3+}H_2}\right]_0 = \frac{k_{NADH} \cdot [NADH]_0 + k_{NADPH} \cdot [NADH]_0) \cdot [HMP_{Fe^{3+}}]_0}{k_{ET} + k_{deg} + d_{HMP_{Fe^{3+}H_2}}} \tag{23}$$

$$[HMP_{Fe^{2+}H}]_0 = \frac{k_{ET} \cdot \left[HMP_{Fe^{3+}H_2}\right]_0 + k_{O_2,OFF} \cdot \left[HMP_{Fe^{2+}HO_2}\right]_0}{k_{deg} + d_{HMP_{Fe^{2+}H}} + k_{O_2,ON} \cdot [O_2]_0} \tag{24}$$

$$[HMP_{Fe^{2+}HO_2}]_0 = \frac{k_{O_2,ON} \cdot [HMP_{Fe^{2+}H}]_0 \cdot [O_2]_0}{k_{deg} + d_{HMP_{Fe^{2+}HO_2}} + k_{O_2,OFF}} \tag{25}$$

Refer to Table S1 for more information on the equations used to derive (21) - (25).

1.7 Fixed metabolite and protein concentrations in growing cells

With the inclusion of growth and the associated dilution of intracellular species in the model, we opted to remove several metabolites and proteins from the mass balance to reflect the fact that these species should not change in concentration as a result of growth or NO treatment (Table S7).

1.8 Growth dependent generation terms for protein and antioxidant species

For proteins and antioxidants that are present in the cell in the absence of NO and are capable of reacting with NO, we assume that the total concentration of these species should remain constant with and without NO treatment. While NO bound and non-NO bound forms of species are able to change in concentration, we assume that the total concentration of all sub-species should remain fixed, regardless of changes in cell density as a result of growth. As such, growth dependent generation terms (f_{gen}) were included to balance out dilution by growth, and thus maintain fixed total concentrations. For a list of species with growth dependent generation terms refer to Table S8.

As an example, for cytochrome *bo* oxidase, total cytochrome *bo* oxidase concentration can be written as the sum of unbound cytochrome *bo* oxidase and NO-bound cytochrome *bo* oxidase:

$$[CYT_{bo_{total}}] = \left[CYT_{bo_{free}}\right] + [CYT_{bo_{NO}}] \tag{26}$$

Taking the derivative of both sides:

$$\frac{d}{dt}[CYT_{bo_{total}}] = \frac{d}{dt}\left[CYT_{bo_{free}}\right] + \frac{d}{dt}[CYT_{bo_{NO}}] \tag{27}$$

The concentration of $[CYT_{bo_{total}}]$ is a constant:

$$0 = \frac{d}{dt}\left[CYT_{bo_{free}}\right] + \frac{d}{dt}[CYT_{bo_{NO}}] \tag{28}$$

Substituting for the rate expressions for free and NO-bound cytochrome:

$$\begin{aligned} 0 = f_{gen} + k_{off} \cdot [CYT_{bo_{NO}}] - k_{on} \cdot [CYT_{bo_{free}}] \cdot [NO] - d_{CYT_{bo_{free}}} \cdot [CYT_{bo_{free}}] \\ +k_{on} \cdot [CYT_{bo_{free}}] \cdot [NO] - k_{off} \cdot [CYT_{bo_{NO}}] - d_{CYT_{bo_{NO}}} \cdot [CYT_{bo_{NO}}] \end{aligned} \tag{30}$$

Solving for f_{gen}:

$$f_{gen} = d_{CYT_{bo_{free}}} \cdot \left[CYT_{bo_{free}}\right] + d_{CYT_{bo_{NO}}} \cdot \left[CYT_{bo_{NO}}\right] \quad (31)$$

Where f_{gen} equals the rate of loss of free and NO bound cytochrome *bo* by dilution in growing cells.

2 Supplementary Figures

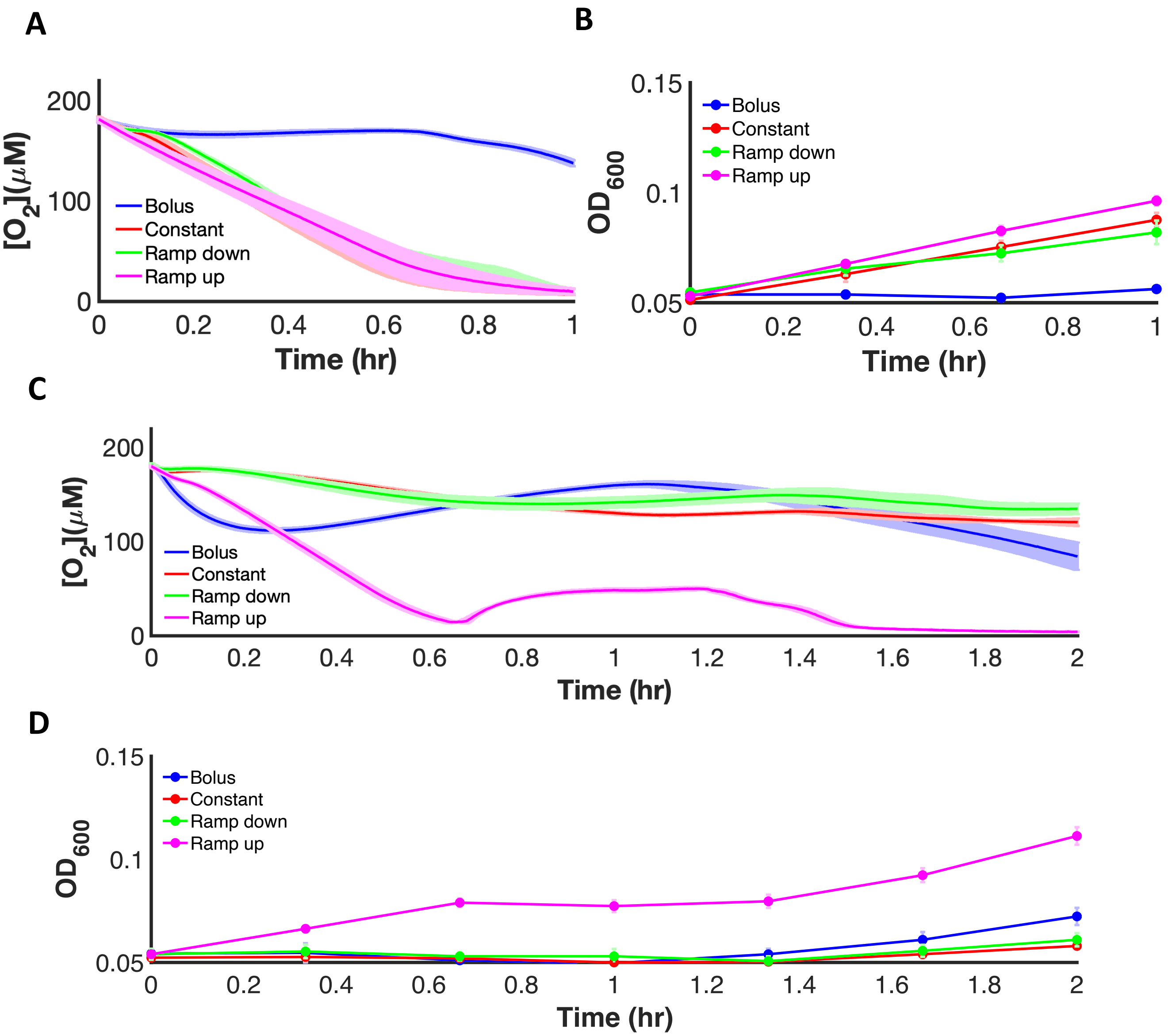

Fig S1. Oxygen and OD_{600} measurements during delivery of 6 and 18 μmol PAPA NONOate. *E.coli* cultures were grown to exponential phase and inoculated in a bioreactor at an OD_{600} of 0.05. Five minutes after inoculation, PAPA NONOate was delivered over an hour in one of four ways (bolus-blue; constant-red; ramp down-green; ramp up-pink) at payloads of 6 μmol (A-B) or 18 μmol (C-D). $[O_2]$ was measured continuously using an optical probe (A & C). Solid lines represent the mean of three independent experiments, whereas the lightly shaded areas represent the standard error of the mean. Cell density was measured by sampling and measuring OD_{600} (B & D). Colored circles represent the mean of three independent experiments, and error bars represent standard error of the mean

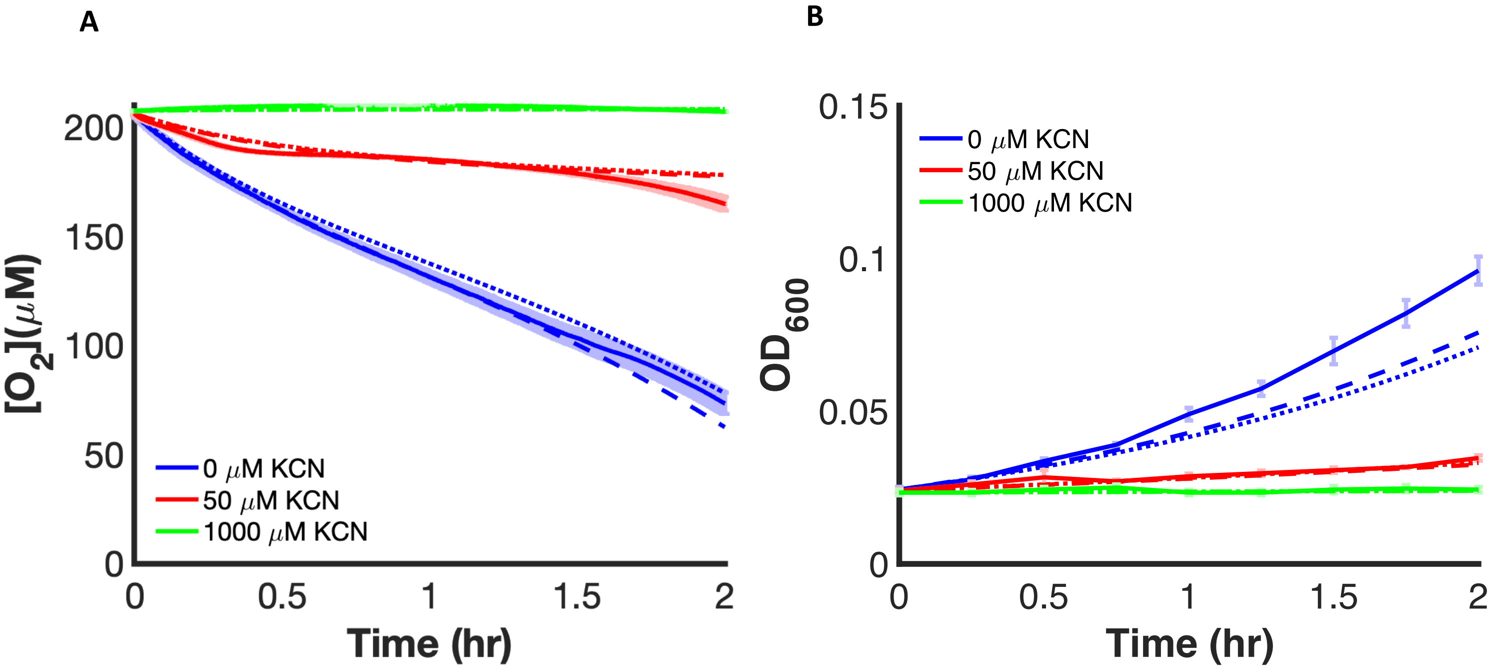

Fig S2. Training parameters related to cellular respiration and growth (refer to Table S5). *E.coli* cultures were grown to exponential phase and inoculated at an OD_{600} of 0.025 into a bioreactor containing 0, 50 or 1000 μM KCN (blue, red and green colors respectively). (A) $[O_2]$ dynamics were continuously measured for 2 hours, while (B) samples were extracted every 30 min to measure OD_{600}. Solid color lines are the mean of 3 independent experiments, whereas the lightly shaded error bars represent the standard error of the mean. 16 parameters were optimized on all six data sets. All parameter sets with ER<10 were retained and considered viable sets. Due to the large size of the ensemble (Figure S11C), only simulations generated from the optimal parameter set (ER=1, minimum SSR, 1 set) and the worst performing member of the ensemble (ER ~ 9.9, largest SSR retained, 1 set) are displayed (dashed and dotted lines respectively).

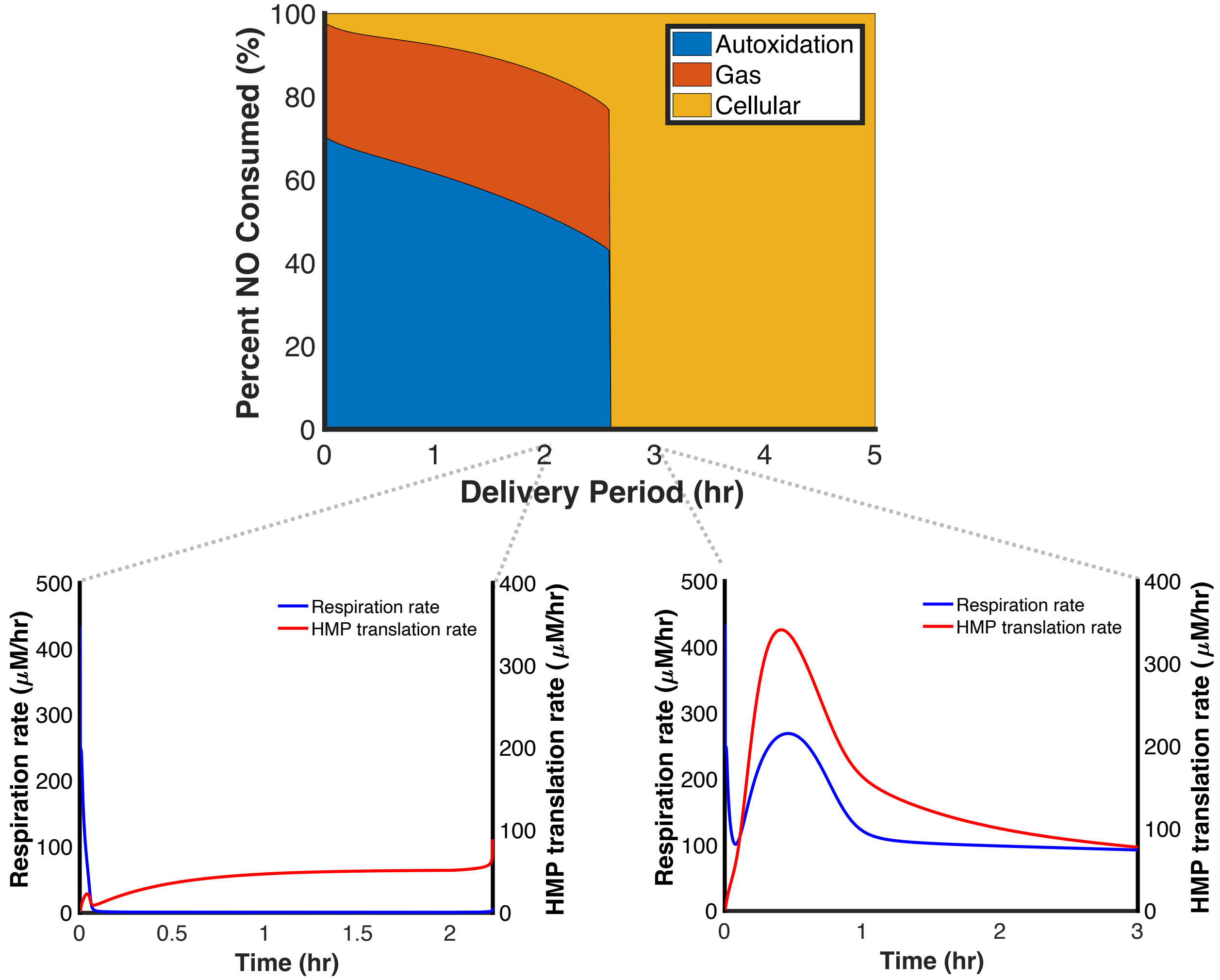

Fig S3. The top panel represents the predicted NO cumulative distribution profiles for constant delivery for 24 μmol PAPA NONOate payload in which delivery periods were varied between 0 and 5 hours. Cumulative [NO] consumed were simulated up to the end of the delivery period or when [NO] dropped below 0.5 μM, whichever was greater. The three major NO consumption pathways are autoxidation (blue), transport to gas phase (red), and cellular consumption (yellow). The left and right panels represent simulations of respiration rates (μM O_2 consumed per hour) (blue) and Hmp translation rates (μM Hmp produced per hour) (red) for delivery periods of 2 hours and 3 hours respectively. All simulations were performed using the optimal parameter set (ER=1, minimum SSR, 1 set).

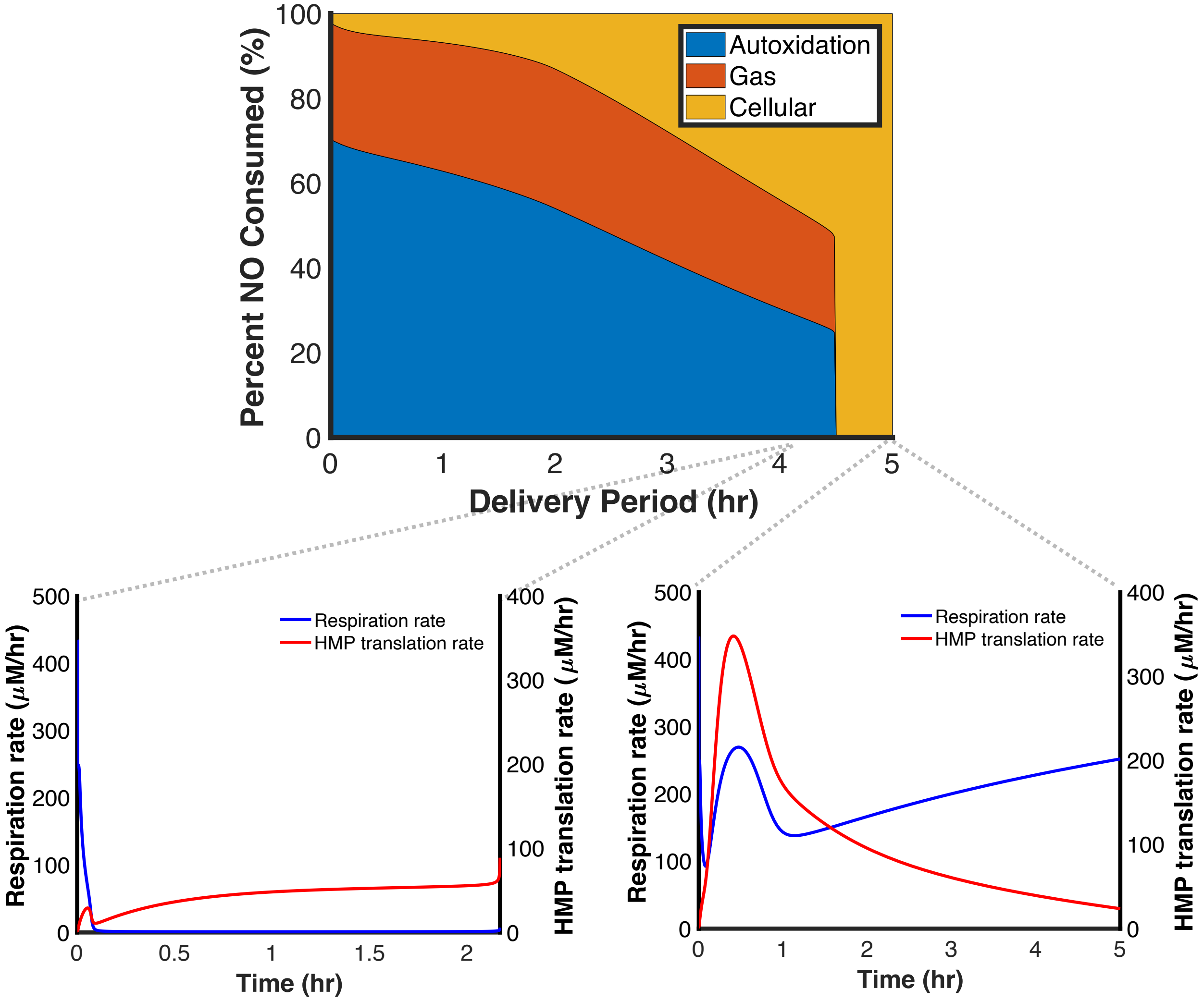

Fig S4. The top panel represents the predicted NO cumulative distribution profiles for ramp down delivery for 24 μmol PAPA NONOate payload in which delivery periods were varied between 0 and 5 hours. Cumulative [NO] consumed were simulated up to the end of the delivery period or when [NO] dropped below 0.5 μM, whichever was greater. The three major NO consumption pathways are autoxidation (blue), transport to gas phase (red), and cellular consumption (yellow). The bottom three panels represent simulations of respiration rates (μM O_2 consumed per hour) (blue) and translation rates (μM Hmp produced per hour) (red) for delivery periods of 0.5, 2 and 4 hours respectively. All simulations were performed using the optimal parameter set (ER=1, minimum SSR, 1 set).

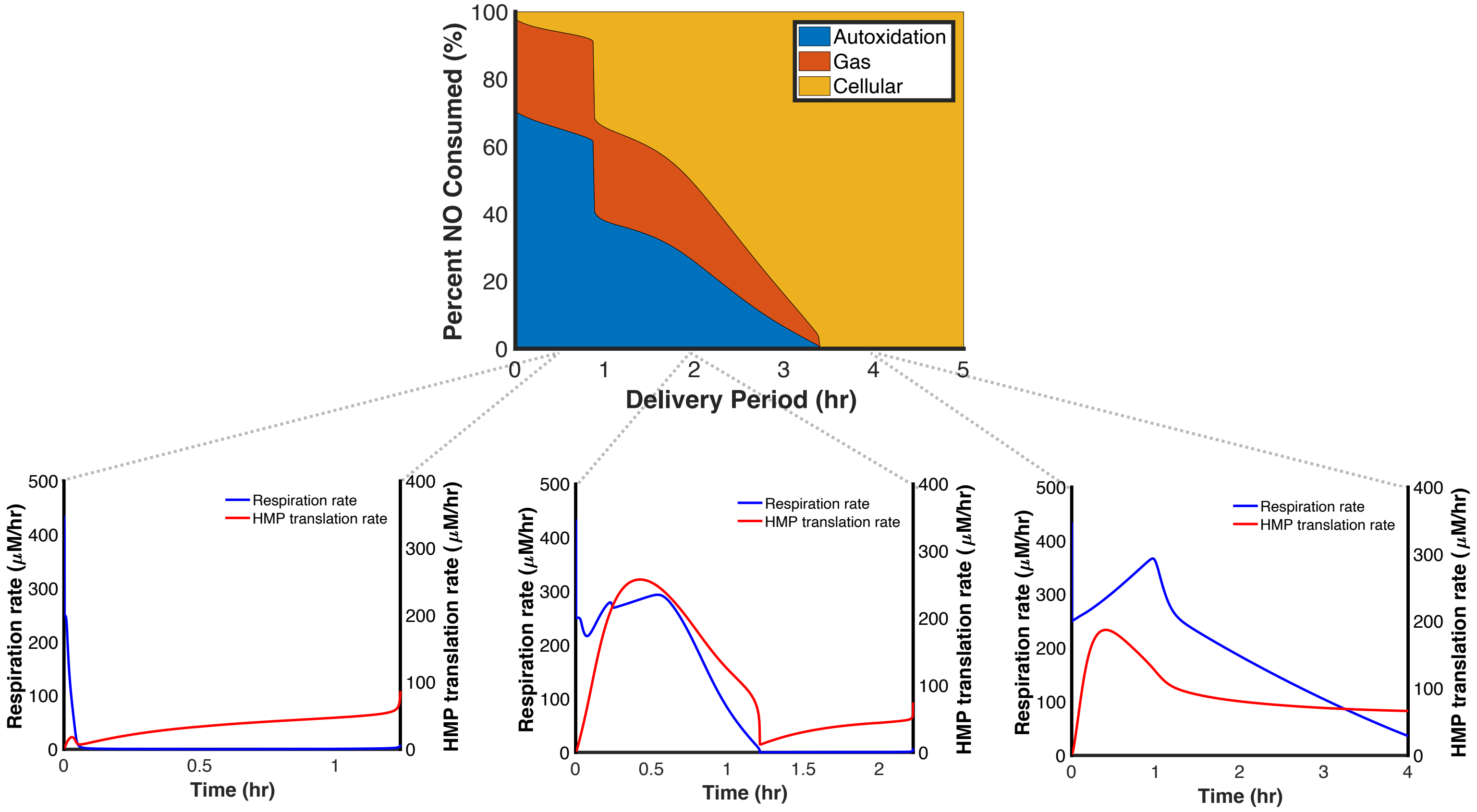

Fig S5. The top panel represents the predicted NO cumulative distribution profiles for ramp up delivery for 24 µmol PAPA NONOate payload in which delivery periods were varied between 0 and 5 hours. Cumulative [NO] consumed were simulated up to the end of the delivery period or when [NO] dropped below 0.5 µM, whichever was greater. The three major NO consumption pathways are autoxidation (blue), transport to gas phase (red), and cellular consumption (yellow). The bottom three panels represent simulations of respiration rates (µM O_2 consumed per hour) (blue) and translation rates (µM Hmp produced per hour) (red) for delivery periods of 0.5, 2 and 4 hours respectively. All simulations were performed using the optimal parameter set (ER=1, minimum SSR, 1 set).

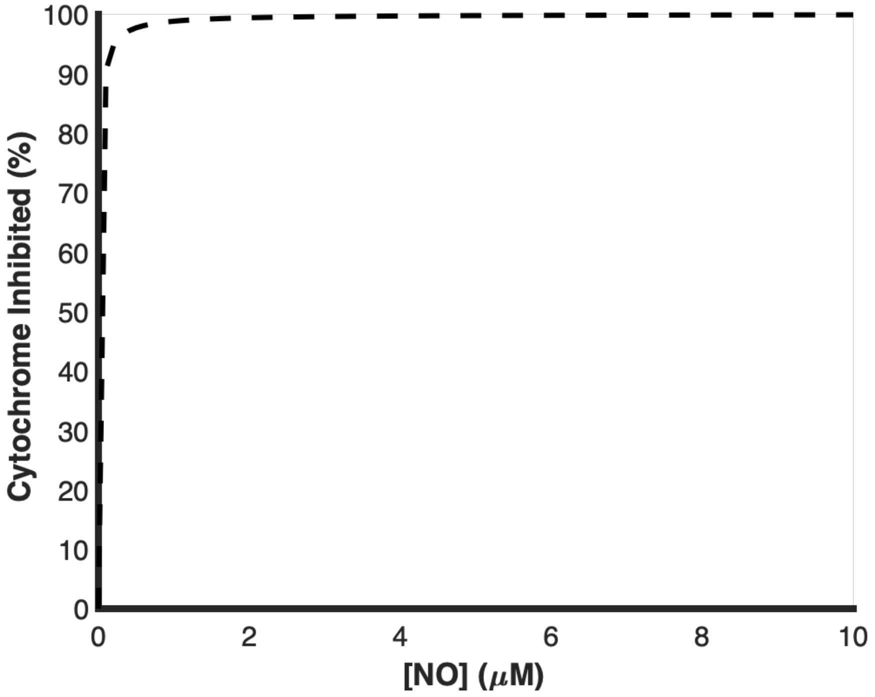

Fig S6. Predicted relationship between [NO] and the percentage of cytochrome inhibited in cultures of *E.coli* at OD_{600} of 0.05. Model simulations were performed using the optimal parameter set (ER=1, minimum SSR, 1 set)

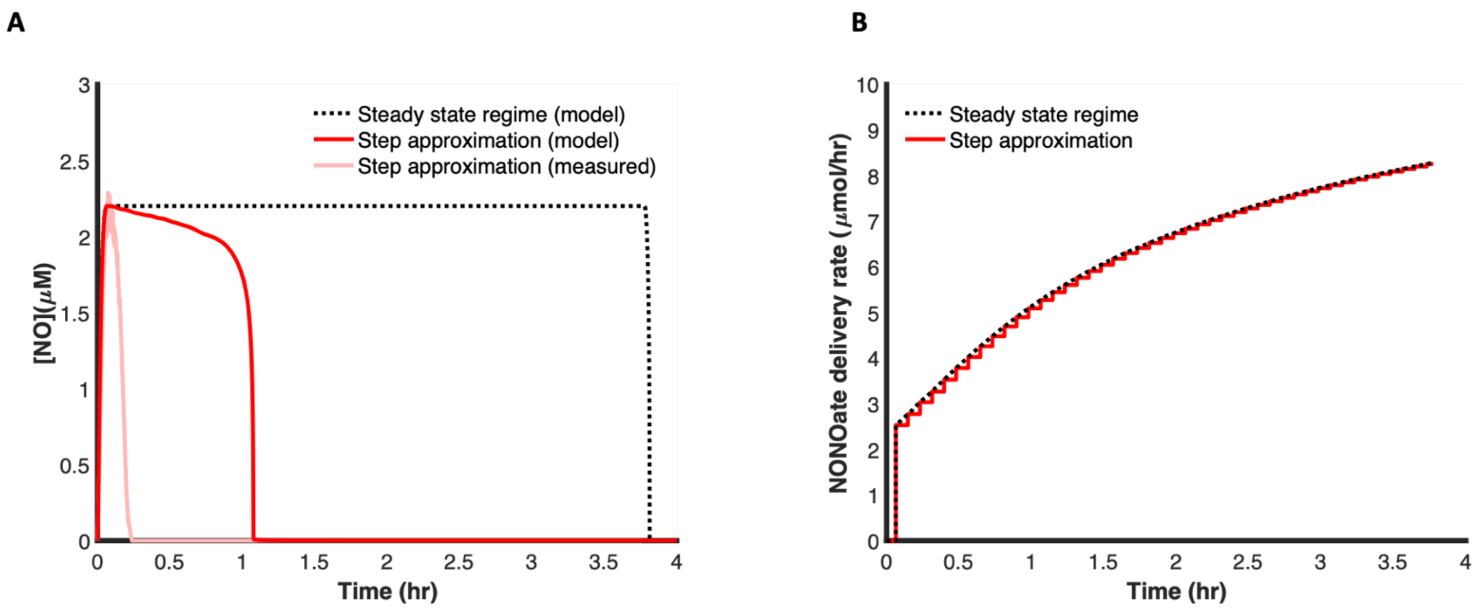

Fig S7. Steady state [NO] simulations. (A) Simulation from the 2.2 μM steady state dosing schedule; dashed black line represents the predicted NO profile using the composite dosing regimen derived from the model. Solid red line represents the measured NO profile using a 5 minute step function approximation to the predicted dosing regimen. Lightly shaded red line represents the measured NO profile using the step approximation. (B) Delivery rate profiles for derived dosing regimen to maintain [NO] at 2.2 μM (black dashed line) and a 5 minute step function approximation (solid red line). All simulations were performed using the optimal parameter set (ER=1, minimum SSR, 1 set).

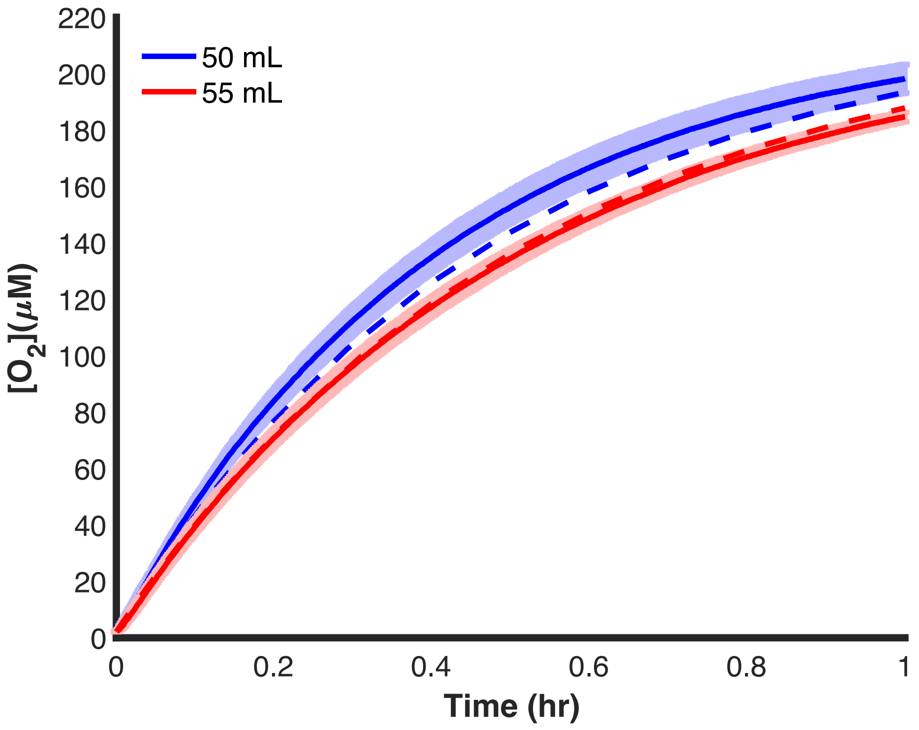

Fig S8. Training of ($k_{LO2} \cdot A$). Oxygen was flushed out of bioreactors containing cell free media with N_2 gas. Following this, [O_2] dynamics were continuously monitored for 1 hour. The process was performed at two different volumes: 50 mL (solid blue line) and 55 mL (solid red line). ($k_{LO2} \cdot A$) was optimized on both curves simultaneously, yielding the [O_2] simulations displayed (dashed blue line for 50 mL; dashed red line for 55 mL) (ER=1, minimum SSR, 1 set). Experimental data are the mean of three independent experiments, whereas the lightly shaded areas represent the standard error of the mean.

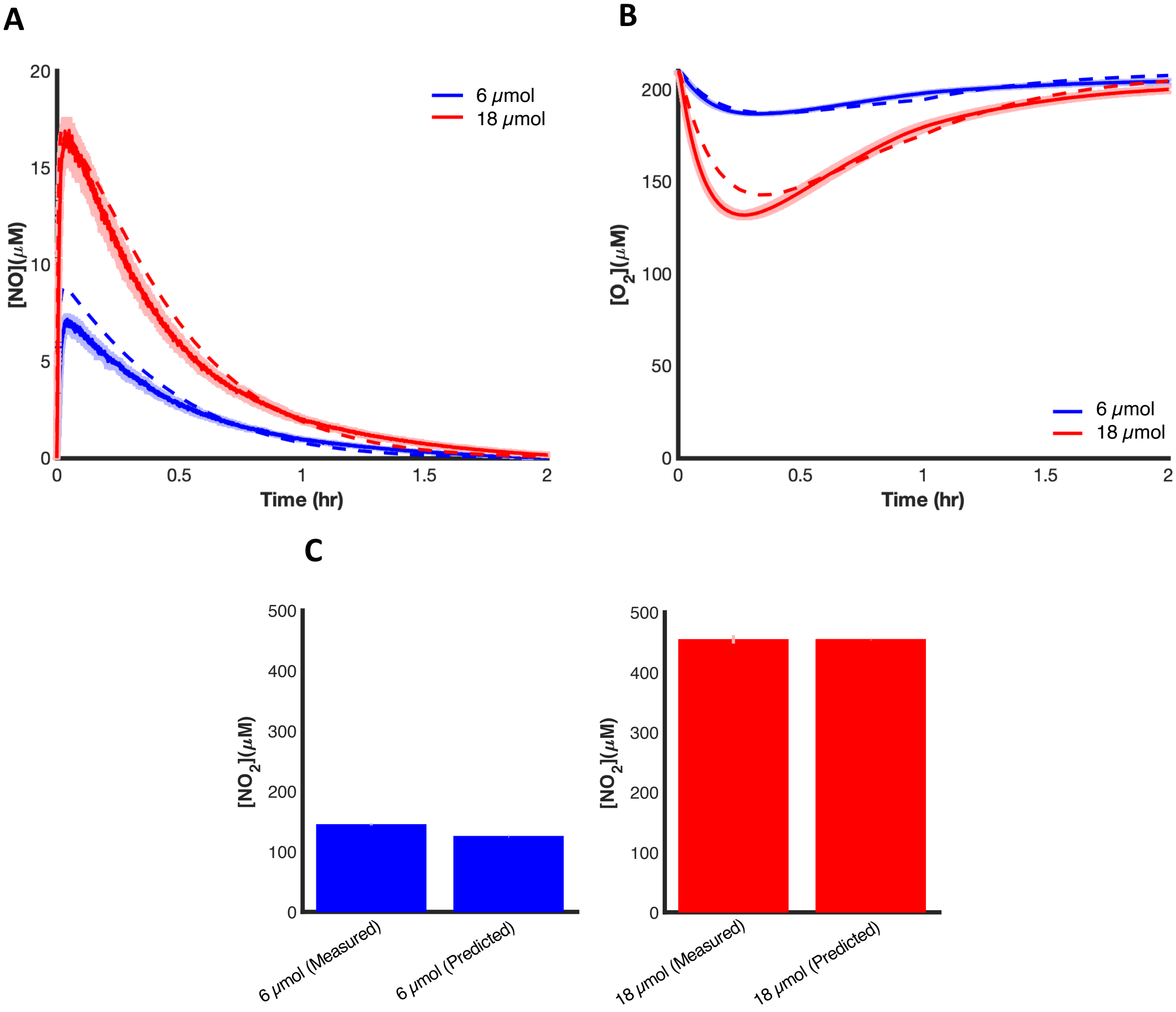

Fig S9. (A-B) Training of extracellular parameters (refer to Table S4). 6 and 18 µmol PAPA NONOate were delivered as boluses to bioreactors containing 50 mL cell-free media. The resulting [NO] and [O_2] dynamics were measured (solid blue and red lines respectively). Extracellular parameters were optimized on all four data sets. All parameter sets with ER<10 were retained and considered viable sets. Due to the large the size of the ensemble and the tight clustering of parameter sets (Figure S11A-B), only simulations generated from the optimal parameter set (ER=1, minimum SSR, 1 set) are displayed (dashed colored lines). (C) The optimal parameter set was used to quantify [NO_2^-] at 2 hours, and simulations were in agreement with measurements. In all cases measured data are the mean of 6 independent experiments, whereas the lightly shaded error bars represent the standard error of the mean.

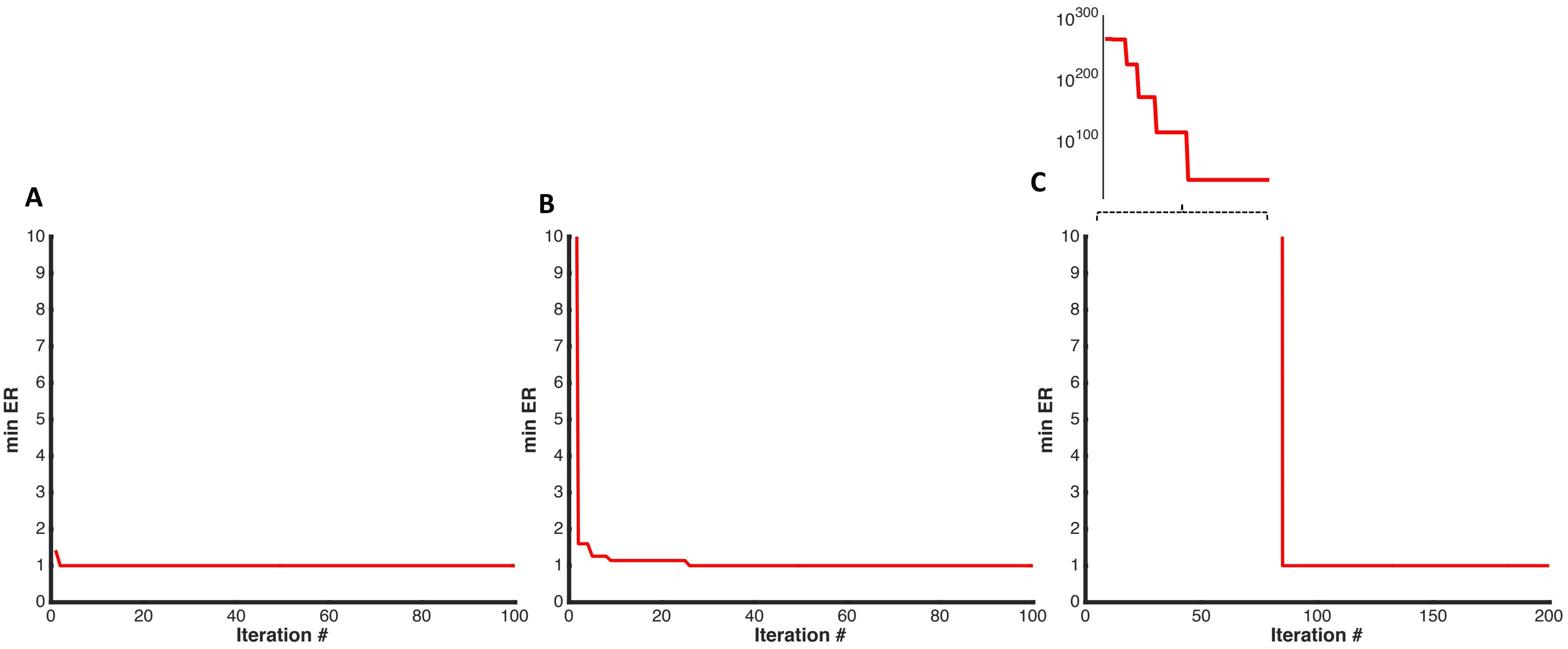

Figure S10. Number of *lsqcurvefit* optimizations performed for (A) extracellular parameters, (B) parameters related to cellular respiration and growth and (C) parameters related to cellular detoxification of NO. Each parameter set optimization was randomly initialized 100 times and evidence ratios (ER) were calculated for each initialization. The number of initializations were capped at 100 if the optimal parameter set (ER=1) was obtained within the first 50 initializations. Otherwise, additional initializations were performed, in batches of 100, until the number of initializations after obtaining the optimal set was greater or equal to the number of initializations needed to obtain the optimal set.

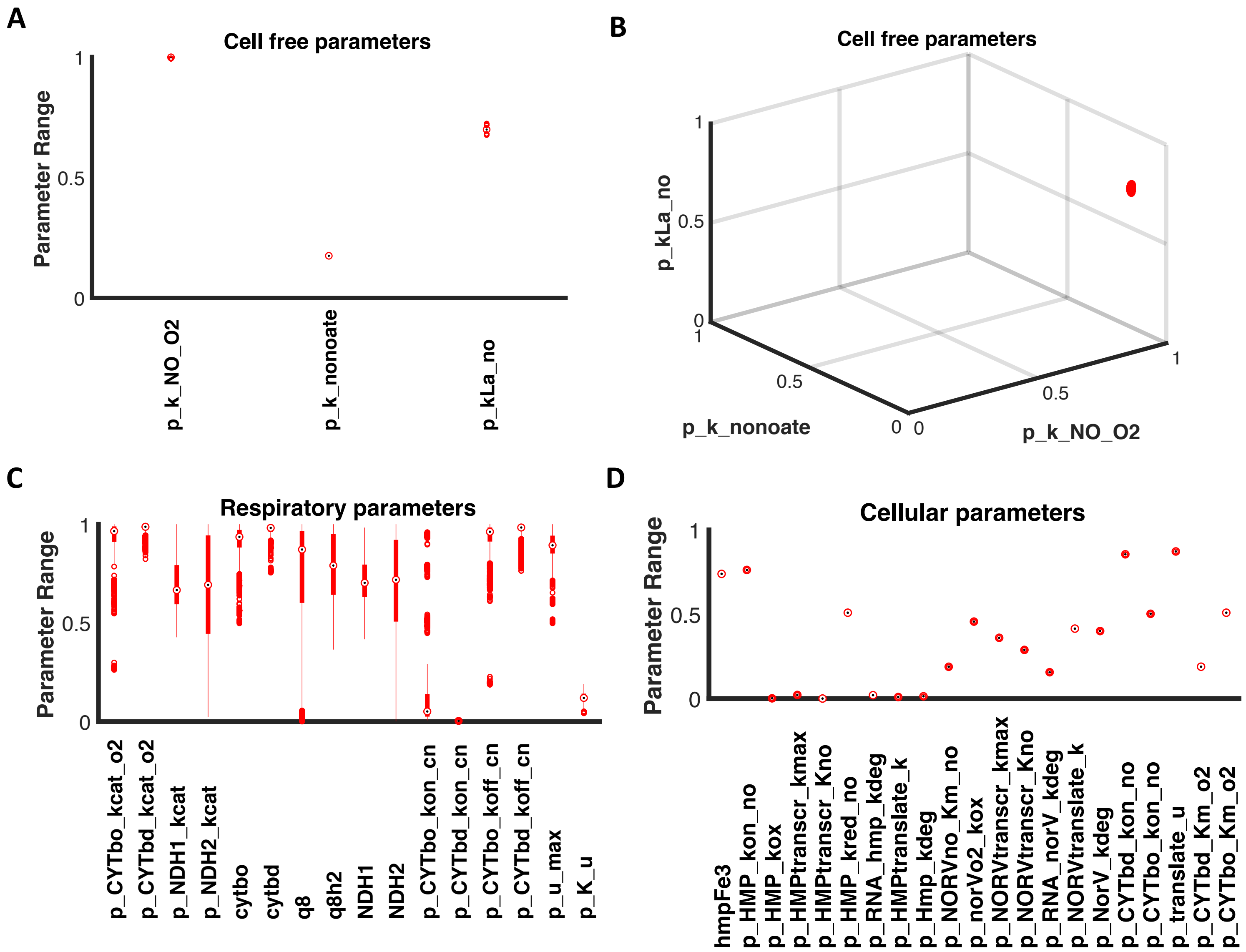

Figure S11. Box plots with normalized parameter ranges (A, C, D) and 3D representation of parameter space (B) for parameter sets within the ensemble (ER<10), following *lsqcurvefit* optimizations and further exploration with the MCMC algorithm. Extracellular parameters: 4294 sets retained (A-B); Respiratory and growth parameters: 17065 sets retained (C); Cellular detoxification parameters: 28 sets retained (D). For box plots, the interquartile range is a red rectangle, the median value is a red circle with a black dot, thin red lines represent box plot whiskers with a length 1.5 times the interquartile range and unfilled circles, often grouped in clusters, represent outliers.

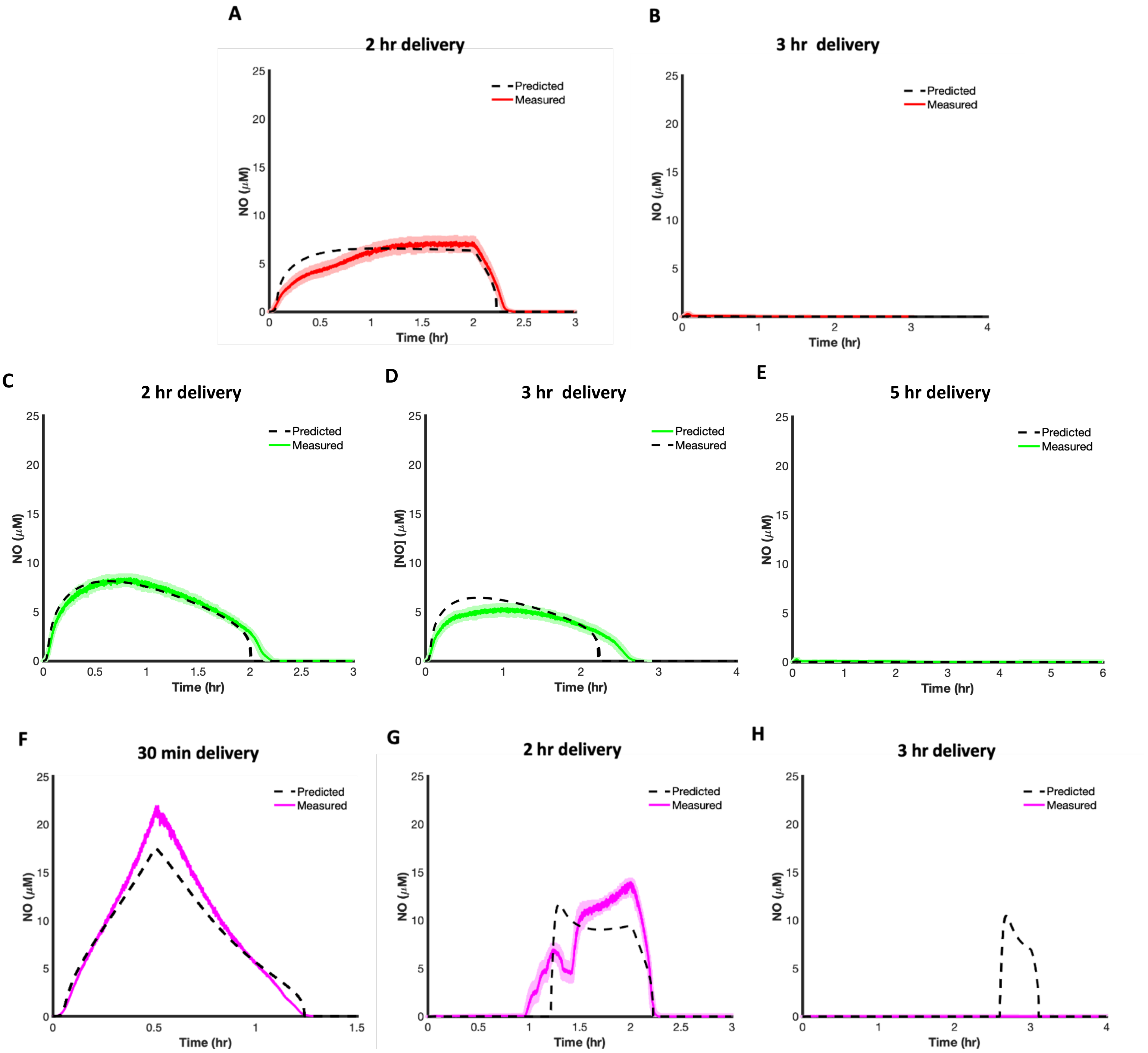

Fig S12. Predicted and measured [NO] dynamics for the delivery of 24 μmol PAPA NONOate over various delivery periods. Cultures of *E.coli* were grown to exponential phase and inoculated in a bioreactor at an OD_{600} of 0.05. Five minutes after inoculation, 24 μmol PAPA NONOate was delivered over the specified delivery period in one of three modes: constant (A-B), ramp down (C-E), or ramp up (F-H). Dashed black lines represent predicted [NO] dynamics using the ensemble of parameter sets (ER<10, 28 sets). The ensemble simulations overlapped to a great extent, thus resembling a single line. Solid colored lines represent the mean of three independent experiments, whereas the lightly shaded areas represent the standard error of the mean.

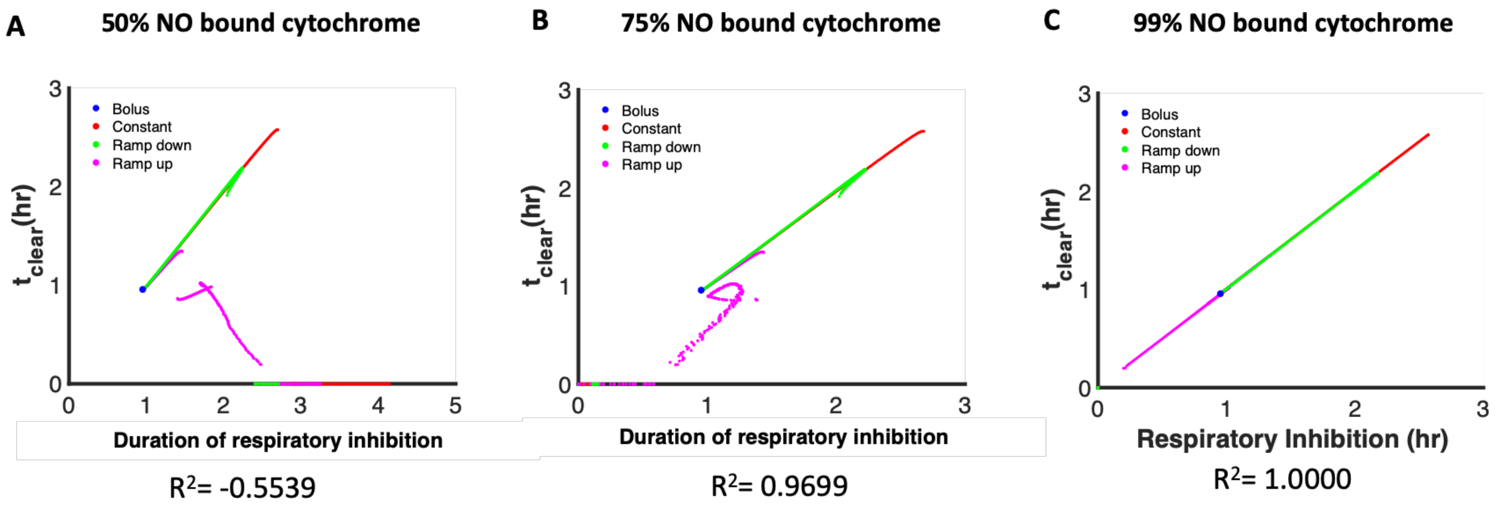

Fig S13. Comparison of duration of respiratory inhibition versus t_{clear} for different metrics of respiratory inhibition: (A) greater or equal to 50% NO bound cytochrome (B) greater or equal to 75% NO bound cytochrome (C) greater or equal to 99% NO bound cytochrome. Simulations were performed using the ensemble of parameter sets (ER<10, 28 sets).

3 Supplementary Tables

Supplementary Tables S1, S2 and S3 are provided as excel files.

Table S4. Extracellular parameter optimization. Parameters relevant to NO autoxidation, NONOate dissociation, and NO gas transport were optimized on [NO] and [O_2] measurements obtained from cell free bioreactors treated with 6 and 18 µmol payloads delivered as a bolus (Fig S9; S11A-B). Values were allowed to vary within bounds, which were from the references provided. Optimal parameter sets were those that yielded the minimum SSR (ER=1). Confidence intervals represent the parameter ranges within the ensemble of sets with ER<10, 4294 sets.

#	Parameter	Parameter Description	Lower Bound	Upper Bound	Optimal	Confidence Interval	Units	Reference
1	'p_k_NO_O2'	NO• autoxidation	0.0032	0.0086	0.0086	0.0086-0.0086	$\mu M^{-2} hr^{-1}$	[2,3,4]
2	'p_k_nonoate'	PAPA NONOate dissocation	0.0347	20.8080	3.687	3.6053-3.7475	hr^{-1}	[5]
3	'p_kLa_no'	NO• loss to the gas phase	0.3600	36.0000	25.4299	24.4316-26.1678	hr^{-1}	[3,4]

Table S5. Cellular respiration and growth parameters optimization. Parameters involved in O_2 respiration and cellular growth were optimized on [O_2] and OD_{600} measurements of exponential phase *E. coli* treated with 0, 50 and 1000 µM KCN (Fig S2; S11C). Values were allowed to vary within bounds, which were from the references provided. Optimal parameter sets were those that yielded the minimum SSR (ER=1). Confidence intervals represent the parameter ranges within the ensemble of sets with ER<10, 17065 sets.

#	Parameter	Parameter Description	Lower Bound	Upper Bound	Optimal	Confidence Interval	Units	Reference
1	'p_CYTbo_kcat_o2'	Cytochrome bo terminal oxidase; kcat	65880	540000	538937.5225	190306.2285 - 539999.7434	hr^{-1}	[6-8]
2	'p_CYTbd_kcat_o2'	Cytochrome bd terminal oxidase; kcat	43200	1688400	1687380.3034	1398023.8087- 1688399.6000	hr^{-1}	[6,8,9]
3	'p_NDH1_kcat'	NADH dehydrogenase I; kcat	180000	2160000	1424655.1823	1025800.9347 - 2159982.9951	hr^{-1}	[10, 11]
4	'p_NDH2_kcat'	NADH dehydrogenase II; kcat	61560	1706400	930143.4068	101382.1604 - 1706359.4713	hr^{-1}	[12,13]
5	'cytbo'	Initial concentration of cytochrome bo	0.0158	1.58	1.5249	0.7943 - 1.5799	µM	[14] a
6	'cytbd'	Initial concentration of cytochrome bd	0.0106	1.06	1.0493	0.8027 - 1.0599	µM	[14] a
7	'q8'	Initial concentration of ubiquinone-8	44.8	4480	2354.6054	46.9638 - 4479.9826	µM	[15] a,b
8	'q8h2'	Initial concentration of ubiquinol-8	44.8	4480	2015.1035	1663.6687 - 4479.9568	µM	[15] a,b
9	'NDH1'	Initial concentration of NADH dehydrogenase I	0.027	2.7	2.0199	1.1416 - 2.6548	µM	[16] a
10	'NDH2'	Initial concentration of NADH dehydrogenase II	0.0031	0.305	0.2230	0.0033 - 0.3049	µM	[17] a
11	'p_CYTbo_kon_cn'	cytochrome bo; inhibitory CN binding	0.036	360000	17850.4286	2394.9868 - 345446.1700	$\mu M^{-1} hr^{-1}$	[18-20] c
12	'p_CYTbd_kon_cn'	cytochrome bd; inhibitory CN binding	0.036	360000	1314.6277	685.5889 - 2064.5382	$\mu M^{-1} hr^{-1}$	[18-20] c
13	'p_CYTbo_koff_cn'	cytochrome bo; inhibitory CN release	0.0036	36000	35876.7039	6731.4205 - 35999.9917	hr^{-1}	[18-20] c
14	'p_CYTbd_koff_cn'	cytochrome bd; inhibitory CN release	0.0036	36000	35269.8170	27495.9324 - 35999.9669	hr^{-1}	[18-20] c
15	'p_u_max'	Maximum specific growth rate	0	1.62	1.5217	0.8069 - 1.6199	hr^{-1}	[21]
16	'p_K_u'	Cellular growth; Kd	0	26.4	3.6704	1.1242 - 5.0435	µM	d

a. Parameter was allowed to vary within 10-fold from the reported value. Data was reported in units molecules/cell, which was then converted to units of concentration assuming a cell volume of 3.2×10^{-15} L [21]
b. Concentrations were reported as 1 µmol/g dry cell weight. Concentrations were estimated by assuming a cell density of 448 gDW/L [16]
c. Approximated from cytochrome c oxidase binding kinetics. Due to the high variability in reported parameters, bounds were allowed to vary within a 100-fold range of the reported values.
d. Upper bound was chosen to be 10-fold higher than the maximum concentrations of cytochrome bo and bd oxidases ($[cytbo]_{max} + [cytbd]_{max}$).

Table S6. NO cellular parameter optimization. Parameters involved in cellular NO detoxification were optimized on [NO] and [O_2] measurements of exponential phase *E. coli* treated with 6 and 18 μmol payloads delivered over 1 hour in each of the principle delivery modes (Fig 3; S11D). Values were allowed to vary within bounds, which were from the references provided. Optimal parameter sets were those that yielded the minimum SSR (ER=1). Confidence intervals represent the parameter ranges within the ensemble of sets with ER<10, 28 sets.

#	Parameter	Parameter Description	Lower Bound	Upper Bound	Optimal	Confidence Interval	Units	Reference
1	'hmpFe3'	Initial concentration of Hmp	0	1.13E-07	8.3740E-08	8.3689E-08 - 8.3795E-08	μM	[22] e
2	'p_HMP_kon_no'	Hmp detoxification; NO• binding to Hmp-Fe2+	14400	93600	74792.0656	74771.5000 - 74816.1346	$\mu M^{-1}hr^{-1}$	[23]
3	'p_HMP_kox'	Hmp detoxification; NO• binding to Hmp-Fe2+-O2	3456000	8640000	3462921.8422	3459847.5350 - 3467706.9076	$\mu M^{-1}hr^{-1}$	[23]
4	'p_HMPtranscr_kmax'	hmp transcription; maximum rate	0.4284	1.6452	0.4542	0.4541 - 0.4548	$\mu M\ hr^{-1}$	[24-26]
5	'p_HMPtranscr_Kno'	hmp transcription; NO• dissociation constant	0.01	10	0.0101	0.0100 - 0.0108	μM	[27, 28]
6	'p_HMP_kred_no'	Hmp detoxification; NO• reduction	46.8	864	462.7136	462.5105- 463.3364	hr^{-1}	[29]
7	'p_RNA_hmp_kdeg'	hmp mRNA degradation	1.206	59.4	2.3727	2.3180 - 2.3802	hr^{-1}	[30, 31]
8	'p_HMPtranslate_k'	Hmp translation	205.2	5364	251.8068	251.4007 - 255.8189	hr^{-1}	[32-35]
9	'p_Hmp_kdeg'	Hmp degradation	0.036	3.6	0.0843	0.0790 - 0.0846	hr^{-1}	[36, 37]
10	'p_NORVno_Km_no'	NorV detoxification; Km,NO•	0.1	1	0.2695	0.2683 - 0.2697	μM	[38, 39]
11	'p_norVo2_kox'	O2-mediated NorV inactivation	0.0036	3600	1642.4288	1638.7951- 1644.3387	$\mu M^{-1}hr^{-1}$	[40, 41]
12	'p_NORVtranscr_kmax'	norV transcription; maximum rate	0.4284	1.6452	0.8667	0.8660- 0.8669	$\mu M\ hr^{-1}$	[24-26]
13	'p_NORVtranscr_Kno'	norV transcription; NO• dissociation constant	0.01	10	2.8909	2.8875 - 2.8995	μM	[27, 28]
14	'p_RNA_norV_kdeg'	norV mRNA degradation	1.206	59.4	10.2896	10.2652 - 10.3220	hr^{-1}	[30, 31]
15	'p_NORVtranslate_k'	NorV translation	205.2	5364	2344.6279	2341.3112 - 2346.8109	hr^{-1}	[32-35]
16	'p_NorV_kdeg'	NorV degradation	0.036	3.6	1.4632	1.4601 - 1.4633	hr^{-1}	[36, 37]
17	'p_CYTbd_kon_no'	cytochrome bd; inhibitory NO• binding	136800	13680000	11719738.0442	11697487.9988 - 11732319.3699	$\mu M^{-1}hr^{-1}$	[42] f
18	'p_CYTbo_kon_no'	cytochrome bo; inhibitory NO• binding	2448	244800	124244.1224	124126.3099 - 124331.0604	$\mu M^{-1}hr^{-1}$	[42] f
19	'p_translate_u'	Growth dependent translation term	0	99	86.4733	86.4313 - 86.5345		g
20	'p_CYTbd_Km_o2'	cytochrome bd respiration; Km,O2	0.027	2.7	0.5317	0.5297 - 0.5321	μM	[42, 43]
21	'p_CYTbo_Km_o2'	cytochrome bo respiration; Km,O2	0.605	60.5	31.0900	31.0776 - 31.1190	μM	[42, 43]

e. The bounds chosen were chosen such that the initial concentration of catalytically active Hmp ($HMPFe^{2+}HO_2$) was restricted to ≤ 1 μM. A reflection of the relatively low levels of Hmp in untreated cells [22]

f. The rate constants for NO binding to cytochromes were permitted to vary within 10-fold of the reported value [42].

g. The bounds were chosen such that protein translation rates were capable of increasing up to 100-fold in growing cells.

Table S7. Model species with fixed concentrations.

#	Species Name	Formula/Abbrev.	Description
1	atp	ATP	Adenosine triphosphate
2	adp	ADP	Adenosine diphosphate
3	amp	AMP	Adenosine monophosphate
4	ala	Ala	L-Alanine
1	co2_air	CO2	Dissoveled carbon dioxide (in equilibrum with air)
5	h	H+	Proton (intracellular)
6	ex_h	H+	Proton (extracellular)
7	h2o	H2O	Water (intracellular)
8	ex_h2o	H2O	Water (extracellular)
9	oh	OH−	hydroxide anion
10	cys	Cys	L-Cysteine
11	datp	dATP	Deoxyadenosine triphosphate
12	dctp	dCTP	Deoxycytosine triphosphate
13	dgtp	dGTP	Deoxyguanosine triphosphate
14	dr5p	dR5P	2-Deoxy-D-ribose 5-phosphate
15	pi	Pi	Phosphate ion
16	ppi	PPi	Diphosphate
17	alka	AlkA	DNA glycosylase (inosine, xanthosine)
18	ung	Ung	DNA glycosylase (uridine)
19	xth	Xth	DNA exonuclease III
20	dnapol	PolI	DNA polymerase I
21	dnalig	LigA	DNA ligase
22	gor	Gor	Glutathione reductase
23	gsfdh	GS-FDH	Glutathione-dependent formaldehyde dehydrogenase
24	nmn	NMN	Nicotinamide mononucleotide
25	nad	NAD+	Nicotinamide adenine dinucleotide (oxidized)
26	nadh	NADH	Nicotinamide adenine dinucleotide (reduced)
27	nadp	NADP+	NAD phosphate (oxidized)
28	nadph	NADPH	NAD phosphate (reduced)
29	o2_air	O2	Dissolved oxygen (in equilibrium with air)
30	fdxox	Fdxox	ferredoxin (oxidized)
31	fdxred	Fdxrd	ferredoxin (reduced)
32	iscs	IscS	Cysteine desulfurase
33	sod	SOD	Superoxide dismutase
34	trp	Trp	L-Tryptophan
35	trxR	TrxR	Thioredoxin reductase
36	tyr	Tyr	L-Tyrosine

Table S8. Model species with growth dependent generation terms in rate expressions.

#	Species Name	Formula/Abbrev.	Description
1	cytbd	Cyd	Cytochrome *bd*
2	cytbo	Cyo	Cytochrome *bo*
3	gsan	GS−	Glutathione (anion)
4	gsh	GSH	Reduced glutathione
5	gssg	GSSG	Oxidized glutathione (disulfide)
6	holo_protein_2fe2s	*P*2Fe2S(*holo*)	Holo [2Fe-2S] protein
7	holo_protein_4fe4s	*P*4Fe4S(*holo*)	Holo [4Fe-4S] protein
8	iscu	IscU	[Fe-S] cluster assembly scaffold protein (dimer)
9	trxrd	Trxred	Thioredoxin (reduced)

Table S9. Sum of squared residuals (SSR), Akaike Information Criteria (AIC), and evidence ratios (ER) for the 28 retained parameter sets retained from the model selection process.

Parameter Set #	SSR	AIC	ER
1	22201.493	4614.944	1.000
2	22208.334	4615.508	1.326
3	22208.440	4615.517	1.332
4	22209.985	4615.644	1.420
5	22212.556	4615.856	1.578
6	22212.633	4615.863	1.583
7	22216.331	4616.168	1.844
8	22221.870	4616.624	2.317
9	22224.226	4616.819	2.553
10	22224.846	4616.870	2.620
11	22225.246	4616.903	2.663
12	22226.518	4617.008	2.806
13	22226.566	4617.012	2.812
14	22228.458	4617.167	3.040
15	22228.692	4617.187	3.070
16	22230.503	4617.336	3.307
17	22235.222	4617.725	4.017
18	22236.027	4617.791	4.152
19	22236.179	4617.804	4.179
20	22237.997	4617.953	4.504
21	22239.399	4618.069	4.771
22	22239.719	4618.095	4.835
23	22240.049	4618.122	4.901
24	22244.183	4618.463	5.810
25	22245.780	4618.595	6.205
26	22254.530	4619.315	8.896
27	22255.660	4619.408	9.320
28	22256.208	4619.453	9.532

References

1. Tse, F. C., and Sandall, O. C. (1979). Diffusion Coefficients For Oxygen And Carbon Dioxide In Water At 25°C By Unsteady State Desorption From A Quiescent Liquid. *Chem. Eng. Comm.* 3, 147–153.
2. Lewis R.S., Deen W.M. (1994). Kinetics of the Reaction of Nitric Oxide with Oxygen in Aqueous Solutions. *Chem. Res. Toxicol.* 7, 568-574.
3. Robinson, J. L., and Brynildsen, M. P. (2016b). Discovery and dissection of metabolic oscillations in the microaerobic nitric oxide response network of *Escherichia coli*. *Proc. Natl. Acad. Sci.* 113, E1757–E1766.
4. Robinson, J. L., and Brynildsen, M. P. (2013). A Kinetic Platform to Determine the Fate of Nitric Oxide in Escherichia coli. *PLoS Comput. Biol.* 9, e1003049.
5. Keefer L.K., Nims R.W., Davies K.M., Wink D.A. (1996). "NONOates" (1-Substituted Diazen-1-ium-1,2-diolates) as Nitric Oxide Donors: Convenient Nitric Oxide Dosage Forms. *Methods. Enzymol.* 268, 281-293.
6. Mason M.G., Shepherd M., Nicholls P., Dobbin P.S., Dodsworth K.S., Poole R.K., Cooper C.E. (2009). Cytochrome bd confers nitric oxide resistance to Escherichia coli. *Nat. Chem. Biol.* 5, 94-96.
7. Bolgiano B., Salmon I., Poole R.K. (1993) Reactions of the membrane-bound cytochrome bo terminal oxidase of Escherichia coli with carbon monoxide and oxygen. *Biochim. Biophys. Acta.*1141, 95-104.
8. Rice C.W., Hempfling W.P. (1978). Oxygen-limited continuous culture and respiratory energy conservation in Escherichia coli. *J Bacteriol.* 134, 115-124.
9. Junemann S., Butterworth P.J., Wrigglesworth J.M. (1995). A suggested mechanism for the catalytic cycle of cytochrome bd terminal oxidase based on kinetic analysis. *Biochemistry.* 34, 14861-14867.
10. Verkhovskaya M.L., Belevich N., Euro L., Wikstrom M., Verkhovsky M.I. (2008). Real-time electron transfer in respiratory complex I. *Proc. Natl. Acad. Sci.* 105:3763-3767.
11. Leif H., Sled V.D., Ohnishi T., Weiss H., Friedrich T. (1995). Isolation and characterization of the proton-translocating NADH: ubiquinone oxidoreductase from Escherichia coli. *Eur. J. Biochem.* 230, 538-548.
12. Jaworowski A., Campbell H.D., Poulis M.I., Young I.G. (1981). Genetic identification and purification of the respiratory NADH dehydrogenase of Escherichia coli. *Biochemistry.* 20, 2041-2047.
13. Villegas J.M., Volentini S.I., Rintoul M.R., Rapisarda V.A. (2011). Amphipathic C-terminal region of Escherichia coli NADH dehydrogenase-2 mediates membrane localization. *Arch. Biochem. Biophys.* 505, 155-159.
14. Cotter P.A., Chepuri V., Gennis R.B., Gunsalus R.P.. (1990). Cytochrome-O (CyoABCDE) and D (CydAB) Oxidase Gene Expression in Escherichia coli Is Regulated by Oxygen, pH, and the Fnr Gene Product. *J. Bacteriol.* 172, 6333-6338.
15. Bekker M., Kramer G., Hartog A.F., Wagner M.J., de Koster C.G., Hellingwerf K.J., de Mattos M.J.T. (2007). Changes in the redox state and composition of the quinone pool of Escherichia coli during aerobic batch-culture growth. *Microbiol.* 153, 1974-1980.
16. Sundararaj S., Guo A., Habibi-Nazhad B., Rouani M., Stothard P., Ellison M., Wishart D.S. (2004). The CyberCell Database (CCDB): a comprehensive, self-

updating, relational database to coordinate and facilitate in silico modeling of Escherichia coli. *Nucleic Acids Res*. 32, D293-D295.

17. Taniguchi Y., Choi P.J., Li G.W., Chen H., Babu M., Hearn J., Emili A., Xie X.S. (2010). Quantifying E. coli proteome and transcriptome with single-molecule sensitivity in single cells. *Science*. 329:533-538.
18. Cooper, C. E., & Brown, G. C. (2008). The inhibition of mitochondrial cytochrome oxidase by the gases carbon monoxide, nitric oxide, hydrogen cyanide and hydrogen sulfide: chemical mechanism and physiological significance. *J. Bioenerg. Biomembr.* 40(5), 533–539.
19. Jones, M. G., Bickar, D., Wilson, M. T., Brunori, M., Colosimo, A., & Sarti, P. (1984). A re-examination of the reactions of cyanide with cytochrome c oxidase. *Biochem. J.* 220(1), 57–66.
20. Erman, J. E. (1974). Kinetic and equilibrium studies of cyanide binding by cytochrome c peroxidase. *Biochemistry,* 13(1), 39–44.
21. Volkmer B., Heinemann M. (2011). Condition-dependent cell volume and concentration of Escherichia coli to facilitate data conversion for systems biology modeling. *PLoS One.* 6, e23126.
22. Poole R.K., Anjum M.F., Membrillo-Hernandez J., Kim S.O., Hughes M.N., et al. (1996). Nitric oxide, nitrite, and Fnr regulation of hmp (flavohemoglobin) gene expression in Escherichia coli K-12. *J. Bacteriol.* 178, 5487-5492.
23. Gardner A.M., Martin L.A., Gardner P.R., Dou Y., Olson J.S. (2000). Steady-state and transient kinetics of Escherichia coli nitric-oxide dioxygenase (flavohemoglobin) - The B10 tyrosine hydroxyl is essential for dioxygen binding and catalysis. *J Biol Chem.* 275, 12581-12589.
24. So L.H., Ghosh A., Zong C., Sepulveda L.A., Segev R., et al. (2011). General properties of transcriptional time series in Escherichia coli. *Nat. Genet.* 43, 554-560.
25. Kennell D., Riezman H. (1977). Transcription and translation initiation frequencies of the Escherichia coli lac operon. *J. Mol. Biol.* 114, 1-21.
26. Liang S., Bipatnath M., Xu Y., Chen S., Dennis P., et al. (1999). Activities of constitutive promoters in Escherichia coli. *J. Mol. Biol.* 292, 19-37.
27. Allen B.W., Liu J., Piantadosi C.A. (2005). Electrochemical detection of nitric oxide in biological fluids. *Methods. Enzymol.* 396, 68-77.
28. Wang C., Trudel L.J., Wogan G.N., Deen W.M. (2003). Thresholds of nitric oxide-mediated toxicity in human lymphoblastoid cells. Chem. Res. Toxicol. 16, 1004-1013.
29. Kim S.O., Orii Y., Lloyd D., Hughes M.N., Poole R.K. (1999). Anoxic function for the Escherichia coli flavohaemoglobin (Hmp): reversible binding of nitric oxide and reduction to nitrous oxide. *FEBS Lett*. 445, 389-394.
30. Bernstein J.A., Khodursky A.B., Lin P.H., Lin-Chao S., Cohen S.N. (2002). Global analysis of mRNA decay and abundance in Escherichia coli at single-gene resolution using two-color fluorescent DNA microarrays. *Proc. Natl. Acad. Sci.* 99, 9697-9702.
31. Selinger D.W., Saxena R.M., Cheung K.J., Church G.M., Rosenow C. (2003). Global RNA half-life analysis in Escherichia coli reveals positional patterns of transcript degradation. *Genome. Res.* 13, 216-223.

32. Sundararaj S., Guo A., Habibi-Nazhad B., Rouani M., Stothard P., et al. (2004). The CyberCell Database (CCDB): a comprehensive, self-updating, relational database to coordinate and facilitate in silico modeling of Escherichia coli. *Nucleic. Acids. Res.* 32, D293-D295.
33. Johansson M., Bouakaz E., Lovmar M., Ehrenberg M. (2008). The kinetics of ribosomal peptidyl transfer revisited. *Mol. Cell.* 30, 589-598.
34. Liang S.T., Xu Y.C., Dennis P., Bremer H. (2000). mRNA composition and control of bacterial gene expression. *J. Bacteriol.* 182, 3037-3044.
35. Bieling P., Beringer M., Adio S., Rodnina M.V. (2006). Peptide bond formation does not involve acid-base catalysis by ribosomal residues. *Nat. Struct. Mol. Biol.* 13, 423-428.
36. Willetts N.S. (1967). Intracellular protein breakdown in growing cells of Escherichia coli. *Biochem. J.* 103, 462-466.
37. Mosteller R.D., Goldstein R.V., Nishimoto K.R. (1980). Metabolism of individual proteins in exponentially growing Escherichia coli. *J. Biol. Chem.* 255, 2524-2532.
38. Vicente J.B., Scandurra F.M., Forte E., Brunori M., Sarti P., Teixeira M., Giuffre A. (2008). Kinetic characterization of the Escherichia coli nitric oxide reductase flavorubredoxin. *Methods. Enzymol.* 437, 47-62.
39. Gardner A., Helmick R., Gardner P. (2002). Flavorubredoxin, an inducible catalyst for nitric oxide reduction and detoxification in Escherichia coli. *J. Biol. Chem.* 277, 8172-8177.
40. Gardner A.M., Gardner P.R. (2002). Flavohemoglobin detoxifies nitric oxide in aerobic, but not anaerobic, Escherichia coli. Evidence for a novel inducible anaerobic nitric oxide-scavenging activity. *J. Biol. Chem.* 277, 8166-8171.
41. Silaghi-Dumitrescu R., Ng K.Y., Viswanathan R., Kurtz D.M., Jr. (2005) A flavo-diiron protein from Desulfovibrio vulgaris with oxidase and nitric oxide reductase activities. Evidence for an in vivo nitric oxide scavenging function. *Biochemistry.* 44, 3572-3579.
42. Mason M.G., Shepherd M., Nicholls P., Dobbin P.S., Dodsworth K.S., Poole R.K., Cooper C.E. (2009). Cytochrome bd confers nitric oxide resistance to Escherichia coli. *Nature. Chem. Biol.* 5, 94-96.
43. Rice C.W., Hempfling W.P. (1978). Oxygen-Limited Continuous Culture and Respiratory Energy Conservation in Escherichia coli. *J. Bacteriol.* 134, 115-124.
